# A systematic review of experiences of advanced practice nursing in general practice

**DOI:** 10.1186/s12912-016-0198-7

**Published:** 2017-01-18

**Authors:** Michael Jakimowicz, Danielle Williams, Grazyna Stankiewicz

**Affiliations:** 1 School of Health Sciences, University of Tasmania, 1 Leichhardt Street, Darlinghurst, NSW 2010 Australia; 2 School of Health Sciences, University of Tasmania, Private Bag 135, Hobart, TAS 7001 Australia; 3 School of Health Sciences, University of Tasmania, Locked Bag 5052, Alexandria, NSW 2015 Australia

**Keywords:** Advanced practice, General practice, Systematic review, Qualitative research

## Abstract

**Background:**

Despite efforts to achieve conceptual clarity, advanced practice nursing continues to reside in a liminal space, unable to secure ongoing recognition as a viable means of healthcare delivery. This is particularly evident in general practice where advanced practice role development is more fluid and generally less supported by the hierarchical structures evident in the hospital system. This review synthesises published qualitative studies reporting experiences of advanced practice nursing in general practice. The panoramic view provided by patients, nurses and doctors within this novel context, offers a fresh perspective on why advanced practice nurses have struggled to gain acceptance within the healthcare milieu.

**Methods:**

We conducted a systematic review of qualitative studies that explored the experiences of patients, nurses and doctors who had contact with advanced practice nurses working in general practice. Published work from 1990 to June 2016 was located using CINAHL and PubMed. The full text of relevant studies was retrieved after reading the title and abstract. Critical appraisal was undertaken and the findings of included studies were analysed using the constant comparative method. Emergent codes were collapsed into sub-themes and themes.

**Results:**

Twenty articles reporting the experiences of 486 participants were included. We identified one major theme: legitimacy; and three sub-themes: (1) establishing and maintaining confidence in the advanced practice nurse, (2) strengthening and weakening boundaries between general practitioners and advanced practice nurses and (3) establishing and maintaining the value of advanced practice nursing.

**Conclusions:**

We set out to describe experiences of advanced practice nursing in general practice. We discovered that general practitioners and patients continue to have concerns around responsibility, trust and accountability. Additionally, advanced practice nurses struggle to negotiate and clarify scopes of practice while general practitioners have trouble justifying the costs associated with advanced practice nursing roles. Therefore, much work remains to establish and maintain the legitimacy of advanced practice nursing in general practice.

**Electronic supplementary material:**

The online version of this article (doi:10.1186/s12912-016-0198-7) contains supplementary material, which is available to authorized users.

## Background

There is an absence of clear agreement regarding the concept of advanced practice nursing both in Australia and overseas [1–5]. Efforts to clarify this uncertainty have concentrated on nomenclature [1], scope [6, 7] and domains of practice [2–5]. We argue that this uncertainty has constrained the transition to unqualified acceptance, wedging advanced practice nursing into a liminal space with little scope for recognition and expansion. This is particularly evident in general practice, where advanced practice role development is more fluid and generally less supported by the hierarchical structures evident in the hospital system [5].

Within the general practice arena, nurses perform advanced practice duties including diabetes education, chronic disease management and mental health casework, supplanting work performed previously by a general practitioner [8–10]. It is generally agreed that this range of responsibilities meets the international expectations of advanced practice nursing in terms of comprehensive care, systems support, education, research and professional leadership [3, 4].

Most research in the general practice area has focused on either (1) Nurse Practitioners, a subset of advance practice nurses with legislative status or (2) Practice Nurses, a larger set of nurses who work in general practice that includes advance practice nurses. A review by McInnes et al. [11] and a study by Merrick et al. [12] provided worthwhile understandings of challenges to teamwork, collaboration and decision-making in the general practice environment without specifically tackling issues surrounding advanced practice nursing. Other studies focused on nurses performing certain roles [13, 14], working in specific contexts [10, 15], or managing particular illnesses [16–18].

The purpose of this review is to synthesise published qualitative studies reporting experiences of advanced practice nursing in general practice. The panoramic view provided by patients, nurses and doctors within this novel context, offers a fresh perspective on why advanced practice nurses have struggled to gain acceptance within the healthcare milieu. This new data will inform wider debates concerning the establishment and continuity of advanced practice roles, independent of setting.

## Methods

### Research question

Our research question was framed using the Population Exposure Outcome (PEO) method as described by Bettany-Saltikov [19]. This framework simplified the search process and facilitated a more focused assessment of the retrieved studies.

We were only interested in the experiences (O) of patients, nurses and doctors (P) who had contact with advanced practice nurses working in general practice (E). We were cognisant of international variations in the use of the term ‘advanced practice’, so we agreed to use Roche et al.’s [5] broader and, therefore, more inclusive definition which was the display of a skill set beyond generic nursing work. While in no way discounting the importance of basic nursing tasks, it was important to establish that advanced practice nursing involved *additional* responsibilities incorporating sophisticated critical reasoning than would not normally be expected of a nurse working at a more junior level. While it could be argued that the intangibility of these higher order skills could lead to errors in recognition, in practice it is relatively straightforward to distinguish what is and what is not advanced practice nursing [1]. In the case of advanced practice nurses working in general practice, this additional work includes, but is not limited to, case management, peer education, chronic disease management, counselling and health promotion. Table 1 provides two examples common to the general practice setting to highlight this delineation.

**Table 1:** Comparison of generic and advanced practice nursing

Nursing task	Generic nursing action	Advanced practice nursing action
Measure blood pressure.	Record result, recognise hypertension, advise general practitioner of result.	Record result, recognise hypertension, obtain patient and family history, discuss treatment options, organise a referral to a general practitioner, discuss the case with the general practitioner in detail, accept responsibility for case management including patient education and further monitoring.
Assess mental status.	Record result, recognise increased agitation, advise general practitioner of situation.	Record result, recognise increased agitation, initiate emergency response if required, use de-escalation techniques developed through formal skills training, organise a referral to a general practitioner, discuss the case with the general practitioner in detail, accept responsibility for ongoing case management including counselling and further monitoring.

Adapted from Dowling et al. [1]

Experiences of the work of a Nurse Practitioner (NP) were also included in the study. Patient participants had to have experience of advanced practice nursing as a current or former patient of a general practice. Nurse participants could be either advanced practice nurses or those being supervised or otherwise interacting with advanced practice nurses. Doctors could be located within the practice in the case of general practitioners (GPs) or extrinsic in the case of specialists.

### Search methods

We located published work from 1990 to June 2016 using the CINAHL and PubMed databases. Preliminary searches revealed that the terms “advanced practice” and “advanced practice nursing” did not capture relevant literature, so we decided to use the broader term of “nursing”. Medical Subject Headings (MeSH) terms for general practice were combined with MeSH terms and text words for nursing and MeSH terms for qualitative research. We limited the search to journal articles in English with the full text available. The search strategies for each database are provided in an additional file [see Additional file 1]. In total, we located 143 studies from PubMed and 45 from CINAHL. Two studies from the authors’ personal collections were also added to this initial group. After discarding duplicate studies, we read the title and abstract of the located articles. Studies reporting survey results needed to include themes derived from “free text” responses. We retrieved the full text of studies that appeared to include relevant data or information. Forty-five studies were retrieved and examined for eligibility.

### Study selection

**Fig. 1:**
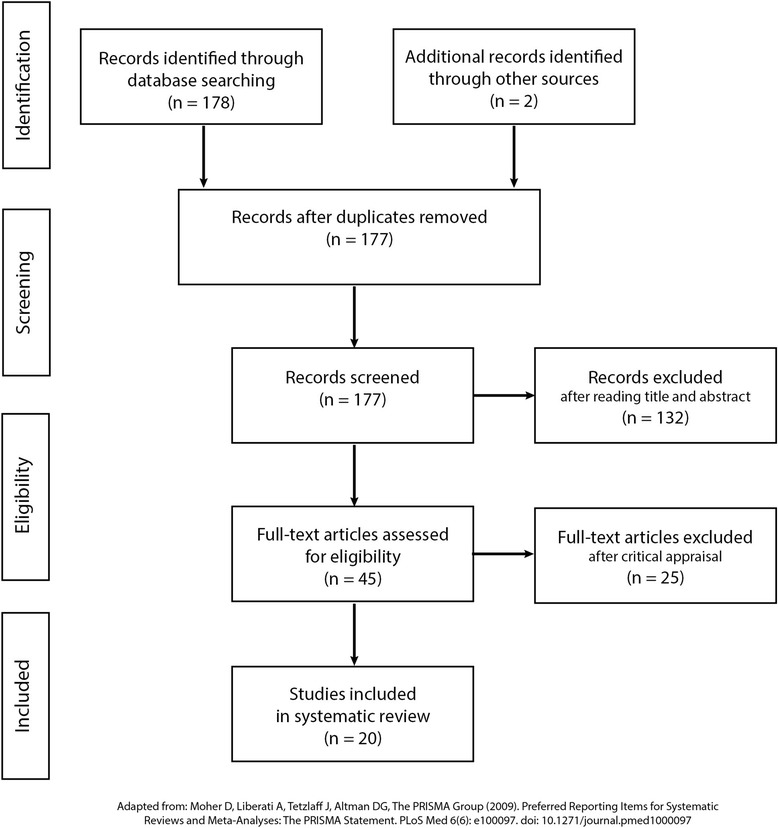
Summary of search process

We developed a critical appraisal tool for this study that focused on methods, analysis and interpretation. The tool was based on the Critical Appraisal Skills Programme (CASP) Checklist for Qualitative Studies [20]. We included an additional screening question that referred to our agreed definition of advanced practice nursing (provided above). Preliminary searches revealed that this step was not feasible during the initial search process. The modified critical appraisal tool is provided in an additional file [see Additional file 2]. Studies were included in the review if questions one, two and three and most of the remaining questions were answered “yes”. Study selection was completed by the lead author and reviewed by the co-authors. The critical appraisal score sheets for each of the included studies is provided in an additional file [see Additional file 3]. The critical appraisal score sheets for the excluded studies is also provided in an additional file [see Additional file 4]. A flow chart describing the results of the search and selection process is provided in Fig. 1.

### Synthesis of findings

The full text of included studies was exported to the NVivo 11^TM^ software program. A list of preliminary codes was developed after close reading of the findings/results section of a selected article. These codes were further refined during analysis of the remaining articles, using the constant comparative method [21]. Text was coded line-by-line and a code tree was used to identify emergent themes. Sub-themes were derived from direct participant quotes and synthesised interpretations. These sub-themes were further analysed and collapsed into one major theme.

## Findings

Twenty articles reporting the experiences of 486 participants were included in the review. Studies were conducted in Australia (10), New Zealand (1), Canada (3), the United Kingdom (5) and continental Europe (1). A summary of these studies is provided in Table 2. The number of participants has been included to highlight the relative weight of patient, nurse and doctor experiences. Overall, we found that there was a paucity of quality studies specifically exploring this phenomenon. Several studies from the United States, a country with a large cohort of advanced practice nurses, were retrieved but subsequently excluded after critical appraisal. Twenty-five studies were excluded in total.

**Table 2:** Studies included in the review

Author(s)	Study location	Methodology and method(s)	Sampling and participants	Phenomena
Blackburn et al. [22]	United Kingdom	Qualitative Semi-structured interviews	Purposive 17 general practitioners (GPs) 17 nurses	Raising the topic of weight
Ehrlich et al. [34]	Australia	Grounded Theory Semi-structured interviews	Purposive 10 GPs 6 nurses	Care coordination
Ehrlich et al. [32]	Australia	Qualitative interpretive Semi-structured focus group interviews	Purposive 9 nurses	Care coordination
Eley et al. [33]	Australia	Mixed Methods Interviews and self-reported questionnaires	Randomised 8 GPs 4 nurses 10 patients	Chronic disease management
Furler et al. [9]	Australia	Qualitative Semi-structured interviews	Purposive 7 GPs 5 nurses 18 patients	Nurse-led model of care for insulin initiation for patients with Type 2 Diabetes Mellitus (T2DM)
Furler et al. [36]	Australia	Qualitative Semi-structured interviews	Purposive 10 GPs 4 diabetes nurse specialists (DNSs) 12 patients	Barriers and enablers to timely initiation of insulin
Johnson and Goyder [26]	United Kingdom	Qualitative Semi-structured interviews	Purposive 12 GPs 3 DNSs 2 nurses	Integrated diabetes care
Mahomed et al. [8]	Australia	Grounded theory In-depth interviews	Purposive 38 patients	The process of patient satisfaction with nurse-led chronic disease management in general practice
Main et al. [30]	United Kingdom	Grounded theory Semi-structured	Purposive 10 GPs 8 nurse practitioners (NPs) 1 practice nurse 2 managers	Barriers to integration of NPs in primary care
Manski-Nankervis et al. [35]	Australia	Qualitative Semi-structured interviews	Purposive 21 GPs, practice nurses and DNSs	The roles and relationships between health professionals involved in insulin initiation
McKenna et al. [24]	Australia	Qualitative Semi-structured interviews	Purposive 23 stakeholders	Barriers and enablers influencing the development of advanced nursing roles in general practice
McKinlay et al. [10]	New Zealand	Qualitative Semi-structured interviews	Unclear 17 nurses	The role of general practice nurses in mental health care
Mills et al. [25]	Australia	Grounded theory Interviews	Purposive 18 nurses	Cervical screening
Mitchell et al. [29]	Canada	Phenomenological Semi-structured interviews	Purposive 16 GPs	NPs as inter-professional educators
Oandasan et al. [23]	Canada	Case study Interviews and focus groups	Purposive 7 nurses	Role and competencies of family practice nurses
Phillips et al. [37]	Australia	Multi-method Interviews, structured observations and artefacts	Illustrative 37 nurses 22 practice managers	Structure and value of nurse and GP labour
Price and Williams [28]	United Kingdom	Qualitative, exploratory Individual interviews and focus groups	Pragmatic 7 NPs 10 GPs 2 nurse lecturers	NP referral practice
Speed and Luker [15]	United Kingdom	Ethnographical Participant observation and semi-structured interviews	Unclear 33 nurses	Methods used by GPs and nurses to organise each other
Sunaert et al. [31]	Belgium	Qualitative Semi-structured interviews and focus groups	Purposive 29 GPs 10 patients	Support to GPs during insulin therapy initiation
Walsh et al. [27]	Canada	Qualitative, descriptive Semi-structured interviews	Purposive 4 NPs 17 medical residents	NPs as educators of medical residents in family practice

**Fig. 2:**
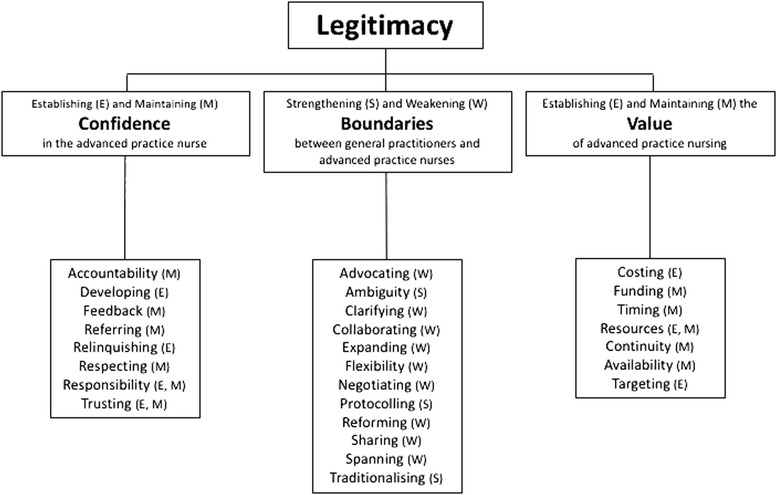
Theme tree

A total of 27 descriptors were used to code the data. We identified three sub-themes: (1) establishing and maintaining confidence in the advanced practice nurse, (2) strengthening and weakening boundaries between general practitioners and advanced practice nurses and (3) establishing and maintaining the value of advanced practice nursing. These were aggregated into one major theme: legitimacy. A diagram showing the relationship of the codes to the sub-themes and major theme is shown in Fig. 2. A list of the studies that contributed to each code and sub-theme is provided in an additional file [see Additional file 5].

### Sub-theme 1 - Establishing and maintaining confidence in the advanced practice nurse

Nineteen out of twenty included studies contributed to this sub-theme [8–10, 15, 22–36]. Of the eight codes used in the aggregation of this sub-theme, the following six codes contributed the greatest amount and are presented below: development, relinquishing, responsibility, trust, accountability and referrals.

#### Establishing confidence in the advanced practice nurse through development

We found that confidence in advanced practice nurses in general practice was established through development activities. Professional development included formal education [22], self-directed learning [23], structured learning pathways [10] and research activities [22]. One study reported that funding of professional activities was an issue [24]. Some nurses were content with their current duties and chose not to participate in further education with a participant stating ‘(we have to) accept that there’s a lot of nurses that don’t want any more responsibility, or they don’t want to extend their roles. They are happy to work within…what they currently do’ [25, p. 3]. Others used the knowledge provided to help them to develop their role further [24, 26]. In some cases, this involved conducting their own development activities within the general practice as part of staff training and mentoring [27–29] and in the community as part of public health programs [25].

We also found a tension between the need to undertake professional development and the drive to establish independent practice. Some nurse practitioners were adamant that professional status would not result from more education, but from a wider scope of practice [30]. Other advanced practice nurses went so far as to claim that skilling less qualified nurses devalued their own training that, in many cases, had cost them thousands of dollars [9]. It was also noted that a minority of GPs still doubted that advanced practice nurses had sufficient education to complete their role [25].

#### General practitioners relinquishing control to display confidence in the advanced practice nurse

We found that over time, GPs became satisfied that they could relinquish certain duties and hand over full or partial responsibility for a range of care activities to advanced practice nurses [28, 31]. This included allowing the advanced practice nurse freedom to operate within their scope of practice [24]. Indeed, GPs considered themselves quite peripheral to the advanced practice nurses in the context of diabetes care, who, they argued had more time to educate patients about glycaemic control [9]. This was also the case with cervical screening, a task that many GPs felt uncomfortable about performing [25]. However, McKinlay et al. [10] found that GPs were reluctant to share the care of mental health patients with advanced practice nurses because it was not an effective use of the nurses’ time. Other GPs were happy to hand off care if they did not have to supervise or otherwise support the advanced practice nurse including answering questions [15, 30]. One nurse stated ‘I think they (doctors) are very happy to leave us to our own devices and I think they are sometimes a little bit unhappy…when we…ask them to look at things we are not happy about and that can cause conflict’ [15, p. 891.]

#### Establishing and maintaining confidence by transferring and accepting responsibility

We found that while many GPs were prepared to handover individual tasks, fewer were willing to assign overall case responsibility to the advanced practice nurse. Two studies found that this only occurred when there were established routines and sustainable structures in place [32, 33]. It was accepted that advanced practice nurses were competent in maintaining the flow of patients through the general practice with one GP stating ‘If she thinks someone needs to be seen, and when, and how, I value that. I follow her advice, and if she disagrees with me then she’ll say so’ [23, p. e379]. However, many GPs did not believe that advanced practice nurses were capable of being both autonomous and accountable [33]. This was reflected in the hesitancy of many nurses to assume full responsibility for patient care [30].

#### Displaying trust to establish and maintain confidence in the advanced practice nurse

We found that trust was an important element in establishing and maintaining confidence in advanced practice nurses in general practice. One study found that trust was the bridge between professional cultures which ultimately benefitted patients [34], while two studies highlighted the importance of medical mentorship in maintaining trust [28, 35]. Diabetic Nurse Educators (DNE) were uniquely placed in this respect because they demonstrated skills regarding insulin initiation and titration that were, in many cases, superior to a GP [35]. The DNE’s relationship with endocrine specialists, allowed GPs to maintain a professional distance that was not replicated with other advanced practice nurses [35]. Another study found that patients needed to see that the DNE carried the authority of, and was trusted by, the GP [36].

Patients also trusted advanced practice nurses who displayed clinical acumen and attitudes by behaving in similar ways to a doctor [23]. Mahomed et al. [8] discovered that patients who had their care needs met were more likely to recognise the level of education, training and experience required to achieve the advanced practice nurse role. A patient in this study stated ‘I presume they’ve all got the same training, they all know what they’re doing and they know what they’ve got to do for me’ [8, p. 2545]. Two studies found that visible and ongoing role development was an essential element of trust [23, 31], while another study found that advanced practice nurses wrestled with the expectation of being both autonomous and a team player with a broad range of professional skills [32].

#### Advanced practice nurses maintaining confidence by accepting accountability

We found that issues around accountability negatively affected confidence in advanced practice nurses in general practice. One study found that while the scope and responsibility of advanced practice was negotiated locally, there was universal agreement between doctors, nurses and patients that the GP was ultimately accountable for decisions made by the nurse [9]. Main et al. [30] found that many NPs were reluctant to fully utilise prescribing rights because they considered themselves to be nurses first and were uncomfortable with being viewed as elitist and acting like a doctor. A NP in this study stated ‘I’m not sure where the resistance emanates from but there’s possibly resistance from the Nursing and Midwifery Council…I think the argument possibly is around the fact that nursing roles are changing so fast that they don’t want to make an elite group’ [30, p. 483]. Senior nurses working towards NP status were in a similar situation [10].

Many patients appreciated having both a GP and an advanced practice nurse involved in their care [33]. Patients reported that the nurse was more likely to ask about any additional concerns that they may have [36]. Advanced practice nurses were also more willing to share information about themselves which put them at ease [8]. This communication style inspired patient confidence in the advanced practice nurse, however, patients did not resonate with approaches that were censorial or dictatorial [8].

#### Doctors maintaining confidence by respecting referrals from advanced practice nurses

We found that some GPs were reluctant to endorse referrals made by NPs to specialists outside the practice on their behalf [28–30, 36]. Some specialists refused to recognise the referral at all and berated the responsible GP for allowing the normal protocol to be bypassed [28]. This gave advanced practice nurses the impression that they were trusted within the walls of the general practice, but not in a way that was visible to the outside world [30]. Of note, was an effort by one advanced practice nurse to reclaim stature by declaring that her role was central to the operation of the practice. She stated ‘we’re, you know running the ship, meaning we’re not able to free up time’ [27, p. e320].

### Sub-theme 2 - Strengthening and weakening boundaries between general practitioners and advanced practice nurses

Every included study contributed to this sub-theme [8–10, 15, 22–37]. Of the 12 codes used in the aggregation of this sub-theme, the following eight codes contributed the greatest amount and are presented below: ambiguity, traditionalising, clarifying, protocolling, reforming, flexibility, collaboration and negotiation.

#### Strengthening the boundary between general practitioners and advanced practice nurses by maintaining the ambiguity of advanced practice nursing roles and responsibilities

One study discovered that despite being supervised by NPs in the initial stages of their training, GP residents were still unclear about the NP’s scope of practice [27]. This uncertainty was also evident in three other studies where more experienced GPs stated that the advanced practice nurse scope of practice was ill-defined and ambiguous [15, 30, 31]. Without a clear understanding of the roles and responsibilities of the advanced practice nurse, some general practitioners lost interest in the position and became disconnected from the advanced practice nurse. This vacuum acted as a boundary between the two areas of practice.

#### Strengthening the boundary between general practitioners and advanced practice nurses by traditionalising doctor-nurse relationships

In an attempt to narrow this gap, some GPs resorted to traditionalising their relationship with the nurse [10, 15, 25, 27, 28, 30–32, 34, 35, 37]. Speed et al. [15] noted incidences of GPs disciplining nurses over the standard of their paperwork. One study reported a case where a GP rationalised role demarcation to who earned the income to pay the nurses [30]. Three studies found that this discordance came down to the initial limited understanding of patients’ needs by the GP [10, 32, 34] which subsequently restricted the capabilities of the advanced practice nurse downstream.

We found that medicalisation of nursing roles was resisted by both nurses and doctors. Some doctors were uncomfortable with nurses making a diagnosis [28] and losing control over treatment decisions [31]. Advanced practice nurses were uneasy with performing time-limited consultations because this reduced the amount of time that they could interact with their patients with one advanced practice nurse stating ‘You are booked solid and you have patient after patient, and when you go back to review they want to talk about all things and you really don’t have time’ [27, p. e320].

#### Strengthening and weakening the boundary between general practitioners and advanced practice nurses through clarifying and protocolling

Clarification both strengthened and weakened the divide between advanced practice nurses and GPs. Within environments that were micro-managed by the GP, seven studies reported that nurses began to doubt their care decisions and sought clarification for increasingly simple matters [10, 15, 28, 30, 31, 34, 36]. One study reported incidences where overt patient requests were overlooked because of this unnecessary interplay between nurse and doctor [34]. Twelve studies reported the development of protocols as a means of avoiding omissions and explicitly stating what duties advanced practice nurses could perform [9, 10, 15, 25, 27, 28, 31–36]. This included computer templates preloaded with pertinent patient information [32], drug initiation and titration algorithms [9, 27] and structured care pathways [10]. In one case a nurse stated ‘…it was tick the boxes, spit out the care plan, spit out the health assessment…we are not dealing with the patient as a holistic person…’ [32, p. 131]. One study reported incidences where GPs believed insulin protocols were only relevant to nurses [31]. Over time, advanced practice nurses lost decision-making skills and felt their status within the practice was devalued [15]. One study found that nurses in this situation preferred to operate within broader policy frameworks [25]. Reassuringly, however, clarification was reported to improve teamwork [30] develop relationships [26] and overcome uncertainty regarding responsibility [31].

#### Weakening the boundary between general practitioners and advanced practice nurses through reforming

Several other behaviours narrowed the gap between GPs and advanced practice nurses. Three studies reported that more experienced GPs were prepared to reform the way care was delivered and expand the role of the advanced practice nurse [25, 28, 32]. One study reported that the ease of implementation was directly correlated with the sustainability of this reorganisation with a GP stating ‘We have to make it easy…we have to make it user friendly’ [32, p. 131]. Another study found that GPs were sceptical of expansion when their own workload pressures increased [31]. One study discovered an interesting tension between DNEs and PNs involving role expansion [35]. In this case, as DNEs became overloaded with additional cases, insulin initiation was delegated to PNs who did not have specialist training and this was viewed as a threat to their status within the practice [35]. Three studies found that nurses had always been trying to expand their roles, citing instances where nurses provided independently organised groups to support patients with chronic illnesses [9, 25, 26]. In another study, patients understood the limitations of care led by advanced practice nurses and realised that they would be referred to a GP if their condition became complicated [8].

#### Weakening the boundary between general practitioners and advanced practice nurses through flexibility

Five studies found that advanced practice nurses and GPs appreciated flexibility [15, 23, 27, 28, 37]. This included balancing multiple priorities [23] and informal communications outside of clinical treatment spaces [37]. A nurse in one study stated ‘It’s a real skill in family practice nursing, identifying those red flags of who needs to be seen – that triaging function’ [23, p. e379]. One study found that a lack of flexibility had serious implications for ongoing relationships [15].

#### Weakening the boundary between general practitioners and advanced practice nurses through collaboration and negotiation

In six studies, advanced practice nurses viewed interactions with GPs as opportunities for collaboration [9, 26, 30, 32, 34, 35]. A further six studies reported negotiation during these exchanges [10, 15, 28, 30, 34, 36]. One study reported that the key components of collaborative relationships were shared knowledge, mutual respect and acceptance [35], while another highlighted the importance of mentoring and supportive networks [32]. In one study, a nurse stated ‘…you do work in isolation. As far as I am aware I am the only primary mental health nurse in (the area)…so I’ve tried to make links with a mental health nursing adviser who provides professional oversight…’ [10, p. 229].

Negotiation was not confined to purely clinical interactions between GPs, advanced practice nurses and patients. One study reported that understanding and utilising key power relationships within the practice, particularly involving those with financial control, was an important skill [32]. Another study found that clinical negotiation skills included overstating a patient’s condition to expedite treatment and challenging/counter-challenging [15]. Interestingly, seven studies reported that patients did not recognise interactions between the advanced practice nurse and the GP as professional cooperation [10, 15, 28, 30, 31, 34, 36]. One patient stated ‘she had to get permission from Dr Ken to put me on insulin, but it was her that decided and he had to say yes’ [9, p. 619].

### Sub-theme 3 - Establishing and maintaining the value of advanced practice nursing

Nineteen out of twenty included studies contributed to this sub-theme [8–10, 15, 22–28, 30–37]. Of the seven codes used in the aggregation of this sub-theme, the following three codes contributed the greatest amount and are presented below: cost, funding and resources.

#### Establishing and maintaining the value of advanced practice nursing by measuring cost, funding and resources

Ten studies reported tensions regarding the cost of advanced practice nurses [9, 10, 24, 25, 30–34, 37]. Another five studies reported anxieties around recouping this expense [9, 24, 31, 32, 34]. Sixteen studies found that as salaried employees, advanced practice nurses measured their worth to the practice in terms of the extra services they offered and the additional time they could give to their patients [8–10, 22, 23, 25–27, 30–37]. One study found that advanced practice nurses believed that the process of establishing a connection with a patient was time consuming in the beginning but reaped dividends in terms of patient compliance [34]. However, a GP in one study stated ‘the nurses can *afford* to spend a little bit more time with the patients than we can’[37, p. 140] implying that a GP’s time was more valuable in dollar terms.

Both nurses and patients reported a reluctance to waste a doctor’s time [37]. One study found that GPs were more conscious of the time versus cost considerations of advanced practice nurses than they were of other services within the general practice, including their own [30]. In another study, a GP believed that NPs were a waste of money because they always asked for a second opinion [22]. In another case, a GP proposed a funding model where patients paid an ‘upfront practice payment’ for services provided by an advanced practice nurse [9, p. 353]. Many GPs believed that it was not their role to provide patient education or engage in health promotion and this task was better left to the advanced practice nurse [15, 24–28, 30, 32, 34, 35, 37]. Some GPs did concede, however, that the advanced practice nurse was an effective means of providing continuity of care to vulnerable patients and many patients suffering chronic illnesses supported this view [8, 9, 24, 25, 28, 30–32, 34].

### Major theme - Legitimacy

The three sub-themes were related by the concept of legitimacy. While GPs, in the main, accepted the place of the advanced practice nurse in the general practice milieu, there was disagreement on how to best utilise this model of care [10, 25–28, 33, 34, 36]. On the one hand, GPs enjoyed handing over what they perceived to be mundane duties to advanced practice nurses, but resented having to pay for the audits that accompanied these tasks [32].

Eleven studies reported that advanced practice nurses stated that they were in a constant battle to be recognised professionally by their colleagues and patients [8, 9, 23, 25, 26, 28, 30–32, 34, 35]. Another study reported that this was also true in relation to other, less qualified, nursing staff [35]. While advanced practice nurses appreciated training opportunities, they struggled to maintain a caseload that was commensurate with their training [31].

Patients, particularly those who viewed their condition as serious, were reluctant to allow an advanced practice nurse to have a prominent role in their care [8]. In two studies, patients viewed the advanced practice nurse as more available than the GP in terms of appointment times, interaction style and spatial positioning within the general practice [23, 33]. While mindful of the need to maintain accessibility, many advanced practice nurses wanted to be accommodated within the general practice in a similar way to the GP, viewing this as a public display of their increased stature within the general practice [15, 22, 27, 28, 31, 32, 34, 37].

## Discussion

### Establishing and maintaining confidence in the advanced practice nurse

The findings showed that advanced practice nurses gained confidence from participation in further training and this assurance was noticeable to colleagues and clients. However, we found that professional development played virtually no part in solidifying the role of the advanced practice nurse within general practices. NPs and DNEs who, by the nature of their position, had more education than other nurses in the practice, believed that the path to recognisable status was increasingly independent practice. They resented PNs being given extended duties after they had completed a relatively small amount of training that was mostly funded by the practice. They also believed that this devalued their on-the-job training and more comprehensive, self-funded education, giving the advanced practice nurses the impression that practice decision makers did not value the nurse’s overall worth to the general practice particularly highly.

We found that there was a tendency for GPs to relinquish duties to advanced practice nurses for reasons other than the skills and abilities of the nurse. This also applied to situations where the GP retained sole responsibility for the task. In many cases, GPs handed over tasks that they had no interest in, did not enjoy performing or took up too much of their consultation time. This created an uneasy tension between GPs and advanced practice nurses because it appeared that GPs were the sole arbiter of what the nurse could or could not do.

We also reported examples where GPs were only happy to handover duties if they were not subsequently called upon by the advanced practice nurse for basic clinical advice, supervision or training. While this could be interpreted that the GP had confidence in the ability of the advanced practice nurse, pragmatically, it meant that tasks of lower clinical importance were delegated. The result of this custom was that advanced practice nurses became unsure of what they were supposed to doing and hesitant to assume additional responsibility when it was offered. Advanced practice nurses were also inclined to default to tasks such as patient flow in the absence of other meaningful work. While important to the day-to-day running of the practice, this task could have been delegated to more junior nurses or indeed reception staff.

Our findings showed that advanced practice nurses were not automatically bestowed with the level of trust that their skills and abilities demanded. It appeared that colleagues either side of the advanced practice nurse, were better placed in this way because they held positions and performed duties that were more easily recognised and understood by patients. To gain respect from GPs, advanced practice nurses felt that they had to display skills that were more medically oriented, however, these skills were not accepted by their less qualified nursing colleagues who themselves felt undervalued and overworked. NPs, who had statutory and nominal advantage over their advanced practice nursing counterparts, still prioritised the nursing component of their practice and were dismayed when their consultations were time restricted.

We found that the concept of accountability was used by both GPs and patients to justify an unwillingness to increase the responsibility of advanced practice nurses. Interestingly, we found that patients, nurses and doctors agreed that the GP was ultimately responsible for a patient’s care in the general practice. While this view could appear to be reasonably justified, today’s healthcare environment demands that every person charged with the care of patients is ultimately answerable for their own practice. Assumptions by GPs that they are responsible for everything that transpires within the practice are, therefore, dangerous because they may give colleagues the (wrong) impression that they are somehow absolved from any culpability deriving from their own care decisions. If patients also expect GPs to retain final say over their care, the advanced practice nurse is, in effect, performing a function that has little relevance. This situation has the potential to create environments where there is a reliance on standing orders and protocols, which only diminishes opportunities for independent practice by advanced practice nurses.

Referral practice was another area of our findings which further exposed the tenuous position of advanced practice nurses in general practice. This traditional view of peer-to-peer referrals is supported by time honoured practices such as referral letters written in standardised, long winded formats that act to exclude newcomers to the arena who do not have a solid grasp of the nuances involved. Given that some GPs also resented advanced practice nurses making diagnoses, it is possible that the pushback from specialists was a means of preserving the last bastion of a closed fraternity.

### Strengthening and weakening boundaries between general practitioners and advanced practice nurses

We found that clarification was both a means of strengthening and weakening boundaries between GPs and advanced practice nurses. In practices where there was a mature relationship between the two, clarification was an empowering force that kept communication channels open and provided opportunities for wider consultation about matters central to the running of the practice. However, other associations were not so productive. In these relationships, advanced practice nurses used clarification as a means of rebuilding their own confidence. This only resulted in trivialising the duties of the advanced practice nurse to the extent that they had to be formalised in a more detailed way with protocols.

### Establishing and maintaining the value of advanced practice nursing

An unexpected finding was the dialectic verbalised by GPs concerning the value of their consultation time versus the recovery of costs incurred through the provision of an advanced practice nurse. We found that on the one hand, GPs were happy to hand over some of the more time-consuming responsibilities of care to nurses to see more patients themselves and, presumably, bring more money into the practice. However, there was a limit to this pattern because nurses are, in the main, salaried from the total earnings of a general practice and recover very little in the way of rebates for their services. This balancing act placed the advanced practice nurse at a considerable disadvantage when compared to a revenue earning GP in terms of justifying their position in the long term. No other studies have identified this tension.

### Legitimacy of advanced practice nursing in general practice

It is clear from our study, that advanced practice nursing does not have a legitimate foothold in general practice. We found that despite patients, nurses and doctors being able to articulate problems concerning confidence, boundaries and value, there had been scant progress towards organising this niche of practice in any sustainable way. Critical theorists such as Willis [38] would claim that this maelstrom is subtly encouraged by the medical profession as a means of asserting and supporting their dominance in the general practice sphere. However, we argue that the uncertainty surrounding advanced practice nursing in general practice is the result of a complex set of related factors that have sabotaged attempts to gain professional recognition for over a decade.

### Implications

Our findings demonstrate that given recent pressures to lower healthcare costs, any attempt to reposition advanced practice nursing as a viable adjunct to medical care will be met with hesitancy by patients and GPs alike. The risk with this paralysis is that without imminent clarification, advanced practice nurses in general practice may be unprepared to accept increasing responsibility as the healthcare burden increases over the next few decades.

### Limitations

We limited this review to qualitative literature to gain a deeper sense of the experiences of advanced practice nursing in general practice. However, many of the included studies did not report participant quotes within the results section of their papers and we, therefore, had to rely on interpretive data for our synthesis. We argue, however, that this does not diminish the ability to generalise our results because all the included studies were subjected to rigorous methodological peer review prior to publication and met our own critical appraisal standards.

## Conclusion

We set out to describe experiences of advanced practice nursing in general practice. We discovered that general practitioners and patients continue to have concerns around responsibility, trust and accountability. Additionally, advanced practice nurses struggle to negotiate and clarify scopes of practice while general practitioners have trouble justifying the costs associated with advanced practice nursing roles. The qualitative literature around advanced practice nursing has shown that this form of nursing has yet to establish sustainable legitimacy in general practice. Given the similarities between this and broader healthcare contexts, we argue that our findings have implications for efforts to solidify advanced practice nursing outside of general practice.

## Additional files

Additional file 1: Search strategies. Description of data: Detailed search strategies for PubMED and CINAHL. (DOCX 36 kb)
Additional file 2: Modified critical appraisal tool. Description of data: Critical appraisal form based on the CASP Checklist for Qualitative Studies. (DOCX 21 kb)
Additional file 3: Critical appraisal of included studies. Description of data: A consolidated document detailing the results of critical appraisal for included studies. (DOCX 70 kb)
Additional file 4: Critical appraisal of excluded studies. Description of data: A consolidated document detailing the results of critical appraisal for excluded studies. (DOCX 80 kb)
Additional file 5 List of studies contributing to each code and sub-theme. Description of data: A table listing the studies that contributed to each code and sub-theme. (DOCX 20 kb)

## Abbreviations

CINAHL: Cumulative Index to Nursing and Allied Health Literature
DNE: Diabetic Nurse Educator
DNS: Diabetic Nurse Specialist
EndNote ^TM^: A reference management software package
GP: General practitioner
MEDLINE: Medical Literature Analysis and Retrieval System Online
MeSH: Medical Subject Heading
NP: Nurse Practitioner
NVivo ^TM^: A qualitative data analysis computer software package
PubMed: A free search engine accessing the MEDLINE database
T2DM: Type 2 Diabetes Mellitus
