# Supplementary material for: A systematic review of experiences of advanced practice nursing in general practice
Source: BMC Nurs. 2017 Jan 18;16:6. doi: 10.1186/s12912-016-0198-7 (PMC5241982; doi:10.1186/s12912-016-0198-7)
Supplement: Additional file 1: — Search strategies. Description of data: Detailed search strategies for PubMED and CINAHL. (DOCX 36 kb) [file 12912_2016_198_MOESM1_ESM.docx]

# Additional file 1: Search strategies

PubMED search strategy:

1 general practice [mh]

2 nurs*[tw] OR jsubsetn[text]

3 qualitative research [mh]

4 1 and 2 and 3

CINAHL search strategy:

S4 S1 AND S2 AND S3

S3 qualitative

S2 nurse

S1 general practice
