# Supplementary material for: A systematic review of experiences of advanced practice nursing in general practice
Source: BMC Nurs. 2017 Jan 18;16:6. doi: 10.1186/s12912-016-0198-7 (PMC5241982; doi:10.1186/s12912-016-0198-7)
Supplement: Additional file 2: — Modified critical appraisal tool. Description of data: Critical appraisal form based on the CASP Checklist for Qualitative Studies. (DOCX 21 kb) [file 12912_2016_198_MOESM2_ESM.docx]

# Additional file 2: Modified critical appraisal tool

**Modified CASP tool**

Reference:

| 1. Was there a clear statement of the aims of the research? | Yes | No | Can’t tell |
| --- | --- | --- | --- |
| 1. Is a qualitative methodology appropriate? | Yes | No | Can’t tell |
| 1. Was there specific mention of advanced practice nursing (per the definition provided in our paper)? | Yes | No | Can’t tell |
| 1. Was the research design appropriate to address the aims of the research? | Yes | No | Can’t tell |
| 1. Was the recruitment strategy appropriate to the aims of the research? | Yes | No | Can’t tell |
| 1. Was the data collected in a way that addressed the research issue? | Yes | No | Can’t tell |
| 1. Has the relationship between researcher and participants been adequately considered? | Yes | No | Can’t tell |
| 1. Have ethical issues been taken into consideration? | Yes | No | Can’t tell |
| 1. Was the data analysis sufficiently rigorous? | Yes | No | Can’t tell |
| 1. Is there a clear statement of findings? | Yes | No | Can’t tell |
| 1. How valuable is the research? | Valuable | Not Valuable |  |

Result:
