# Supplementary material for: A systematic review of experiences of advanced practice nursing in general practice
Source: BMC Nurs. 2017 Jan 18;16:6. doi: 10.1186/s12912-016-0198-7 (PMC5241982; doi:10.1186/s12912-016-0198-7)
Supplement: Additional file 3: — Critical appraisal of included studies. Description of data: A consolidated document detailing the results of critical appraisal for included studies. (DOCX 70 kb) [file 12912_2016_198_MOESM3_ESM.docx]

# Additional file 3: Critical appraisal of included studies

**Modified CASP tool**

Reference: Blackburn M, Stathi A, Keogh E, Eccleston C: Raising the topic of weight in general practice: perspectives of GPs and primary care nurses. *BMJ Open* 2015, 5(8):e008546.

| 1. Was there a clear statement of the aims of the research? | Yes | No | Can’t tell |
| --- | --- | --- | --- |
| 1. Is a qualitative methodology appropriate? | Yes | No | Can’t tell |
| 1. Was there specific mention of advanced practice nursing (per the definition provided in our paper)? | Yes | No | Can’t tell |
| 1. Was the research design appropriate to address the aims of the research? | Yes | No | Can’t tell |
| 1. Was the recruitment strategy appropriate to the aims of the research? | Yes | No | Can’t tell |
| 1. Was the data collected in a way that addressed the research issue? | Yes | No | Can’t tell |
| 1. Has the relationship between researcher and participants been adequately considered? | Yes | No | Can’t tell * |
| 1. Have ethical issues been taken into consideration? | Yes | No | Can’t tell |
| 1. Was the data analysis sufficiently rigorous? | Yes | No | Can’t tell |
| 1. Is there a clear statement of findings? | Yes | No | Can’t tell |
| 1. How valuable is the research? | Valuable | Not Valuable |  |

Result: INCLUDED *No mention of researcher’s own role, potential bias and influence.

**Modified CASP tool**

Reference: Ehrlich C, Kendall E, Muenchberger H: **Spanning boundaries and creating strong patient relationships to coordinate care are strategies used by experienced chronic condition care coordinators**. *Contemporary Nurse* 2012, **42**(1):67-75.

| 1. Was there a clear statement of the aims of the research? | Yes | No | Can’t tell |
| --- | --- | --- | --- |
| 1. Is a qualitative methodology appropriate? | Yes | No | Can’t tell |
| 1. Was there specific mention of advanced practice nursing (per the definition provided in our paper)? | Yes | No | Can’t tell |
| 1. Was the research design appropriate to address the aims of the research? | Yes | No | Can’t tell |
| 1. Was the recruitment strategy appropriate to the aims of the research? | Yes | No | Can’t tell |
| 1. Was the data collected in a way that addressed the research issue? | Yes | No | Can’t tell |
| 1. Has the relationship between researcher and participants been adequately considered? | Yes | No | Can’t tell |
| 1. Have ethical issues been taken into consideration? | Yes | No | Can’t tell |
| 1. Was the data analysis sufficiently rigorous? | Yes | No | Can’t tell |
| 1. Is there a clear statement of findings? | Yes | No | Can’t tell |
| 1. How valuable is the research? | Valuable | Not Valuable |  |

Result: INCLUDED

**Modified CASP tool**

Reference: Ehrlich C, Kendall E, St John W: **How does care coordination provided by registered nurses "fit" within the organisational processes and professional relationships in the general practice context?** *Collegian (Royal College of Nursing, Australia)* 2013, **20**(3):127-135.

| 1. Was there a clear statement of the aims of the research? | Yes | No | Can’t tell |
| --- | --- | --- | --- |
| 1. Is a qualitative methodology appropriate? | Yes | No | Can’t tell |
| 1. Was there specific mention of advanced practice nursing (per the definition provided in our paper)? | Yes | No | Can’t tell |
| 1. Was the research design appropriate to address the aims of the research? | Yes | No | Can’t tell |
| 1. Was the recruitment strategy appropriate to the aims of the research? | Yes | No | Can’t tell |
| 1. Was the data collected in a way that addressed the research issue? | Yes | No | Can’t tell |
| 1. Has the relationship between researcher and participants been adequately considered? | Yes | No | Can’t tell * |
| 1. Have ethical issues been taken into consideration? | Yes | No | Can’t tell |
| 1. Was the data analysis sufficiently rigorous? | Yes | No | Can’t tell |
| 1. Is there a clear statement of findings? | Yes | No | Can’t tell |
| 1. How valuable is the research? | Valuable | Not Valuable |  |

Result: INCLUDED *No mention of researcher’s own role, potential bias and influence.

**Modified CASP tool**

Reference: Eley E, Patterson E, Young J, Fahey P, Del Mar C, Hegney D, Synnott R, Mahomed R, Baker P, Scuffham P: **Outcomes and opportunities: a nurse-led model of chronic disease management in Australian general practice**. *Australian Journal of Primary Health* 2013, **19**:150-158.

| 1. Was there a clear statement of the aims of the research? | Yes | No | Can’t tell |
| --- | --- | --- | --- |
| 1. Is a qualitative methodology appropriate? | Yes  ^1^ | No | Can’t tell |
| 1. Was there specific mention of advanced practice nursing (per the definition provided in our paper)? | Yes | No | Can’t tell |
| 1. Was the research design appropriate to address the aims of the research? | Yes | No | Can’t tell |
| 1. Was the recruitment strategy appropriate to the aims of the research? | Yes | No | Can’t tell |
| 1. Was the data collected in a way that addressed the research issue? | Yes | No | Can’t tell |
| 1. Has the relationship between researcher and participants been adequately considered? | Yes | No | Can’t tell ^2^ |
| 1. Have ethical issues been taken into consideration? | Yes | No | Can’t tell |
| 1. Was the data analysis sufficiently rigorous? | Yes | No | Can’t tell |
| 1. Is there a clear statement of findings? | Yes | No | Can’t tell |
| 1. How valuable is the research? | Valuable | Not Valuable |  |

Result: INCLUDED 1. Mixed Methods 2. No mention of researcher’s own role, potential bias and influence.

**Modified CASP tool**

Reference: Furler J, Blackberry I, Walker C, Manski-Nankervis J, Anderson J, O'Neal D, Young D, Best J: Stepping up: a nurse-led model of care for insulin initiation for people with type 2 diabetes. *Family Practice* 2014, 31(3):349-356.

| 1. Was there a clear statement of the aims of the research? | Yes | No | Can’t tell |
| --- | --- | --- | --- |
| 1. Is a qualitative methodology appropriate? | Yes | No | Can’t tell |
| 1. Was there specific mention of advanced practice nursing (per the definition provided in our paper)? | Yes | No | Can’t tell |
| 1. Was the research design appropriate to address the aims of the research? | Yes | No | Can’t tell |
| 1. Was the recruitment strategy appropriate to the aims of the research? | Yes | No | Can’t tell |
| 1. Was the data collected in a way that addressed the research issue? | Yes | No | Can’t tell |
| 1. Has the relationship between researcher and participants been adequately considered? | Yes | No | Can’t tell * |
| 1. Have ethical issues been taken into consideration? | Yes | No | Can’t tell |
| 1. Was the data analysis sufficiently rigorous? | Yes | No | Can’t tell |
| 1. Is there a clear statement of findings? | Yes | No | Can’t tell |
| 1. How valuable is the research? | Valuable | Not Valuable |  |

Result: INCLUDED *No mention of researcher’s own role, potential bias and influence.

**Modified CASP tool**

Reference: Furler J, Spitzer O, Young D, Best J: Insulin in general practice - barriers and enablers for timely initiation. *Australian Family Physician* 2011, 40(8):617-621.

| 1. Was there a clear statement of the aims of the research? | Yes | No | Can’t tell |
| --- | --- | --- | --- |
| 1. Is a qualitative methodology appropriate? | Yes | No | Can’t tell |
| 1. Was there specific mention of advanced practice nursing (per the definition provided in our paper)? | Yes | No | Can’t tell |
| 1. Was the research design appropriate to address the aims of the research? | Yes | No | Can’t tell |
| 1. Was the recruitment strategy appropriate to the aims of the research? | Yes | No | Can’t tell |
| 1. Was the data collected in a way that addressed the research issue? | Yes | No | Can’t tell |
| 1. Has the relationship between researcher and participants been adequately considered? | Yes | No | Can’t tell |
| 1. Have ethical issues been taken into consideration? | Yes | No | Can’t tell |
| 1. Was the data analysis sufficiently rigorous? | Yes | No | Can’t tell |
| 1. Is there a clear statement of findings? | Yes | No | Can’t tell |
| 1. How valuable is the research? | Valuable | Not Valuable |  |

Result: INCLUDED

**Modified CASP tool**

Reference: Johnson M, Goyder E: Changing roles, changing responsibilities and changing relationships: an exploration of the impact of a new model for delivering integrated diabetes care in general practice. *Quality in Primary Care* 2005, 13(2):85-90.

| 1. Was there a clear statement of the aims of the research? | Yes | No | Can’t tell |
| --- | --- | --- | --- |
| 1. Is a qualitative methodology appropriate? | Yes | No | Can’t tell |
| 1. Was there specific mention of advanced practice nursing (per the definition provided in our paper)? | Yes | No | Can’t tell |
| 1. Was the research design appropriate to address the aims of the research? | Yes | No | Can’t tell |
| 1. Was the recruitment strategy appropriate to the aims of the research? | Yes | No | Can’t tell |
| 1. Was the data collected in a way that addressed the research issue? | Yes | No | Can’t tell |
| 1. Has the relationship between researcher and participants been adequately considered? | Yes | No | Can’t tell * |
| 1. Have ethical issues been taken into consideration? | Yes | No | Can’t tell |
| 1. Was the data analysis sufficiently rigorous? | Yes | No | Can’t tell |
| 1. Is there a clear statement of findings? | Yes | No | Can’t tell |
| 1. How valuable is the research? | Valuable | Not Valuable |  |

Result: INCLUDED *No mention of researcher’s own role, potential bias and influence.

**Modified CASP tool**

Reference: Mahomed R, St John W, Patterson E: Understanding the process of patient satisfaction with nurse-led chronic disease management in general practice. *Journal of Advanced Nursing* 2012, 68(11):2538-2549.

| 1. Was there a clear statement of the aims of the research? | Yes | No | Can’t tell |
| --- | --- | --- | --- |
| 1. Is a qualitative methodology appropriate? | Yes | No | Can’t tell |
| 1. Was there specific mention of advanced practice nursing (per the definition provided in our paper)? | Yes | No | Can’t tell |
| 1. Was the research design appropriate to address the aims of the research? | Yes | No | Can’t tell |
| 1. Was the recruitment strategy appropriate to the aims of the research? | Yes | No | Can’t tell |
| 1. Was the data collected in a way that addressed the research issue? | Yes | No | Can’t tell |
| 1. Has the relationship between researcher and participants been adequately considered? | Yes | No | Can’t tell * |
| 1. Have ethical issues been taken into consideration? | Yes | No | Can’t tell |
| 1. Was the data analysis sufficiently rigorous? | Yes | No | Can’t tell |
| 1. Is there a clear statement of findings? | Yes | No | Can’t tell |
| 1. How valuable is the research? | Valuable | Not Valuable |  |

Result: INCLUDED *No mention of researcher’s own role, potential bias and influence.

**Modified CASP tool**

Reference: Main R, Dunn N, Kendall K: **Crossing professional boundaries': barriers to the integration of nurse practitioners in primary care**. *Education for Primary Care* 2007, **18**(4):480-487.

| 1. Was there a clear statement of the aims of the research? | Yes | No | Can’t tell |
| --- | --- | --- | --- |
| 1. Is a qualitative methodology appropriate? | Yes | No | Can’t tell |
| 1. Was there specific mention of advanced practice nursing (per the definition provided in our paper)? | Yes | No | Can’t tell |
| 1. Was the research design appropriate to address the aims of the research? | Yes | No | Can’t tell |
| 1. Was the recruitment strategy appropriate to the aims of the research? | Yes | No | Can’t tell |
| 1. Was the data collected in a way that addressed the research issue? | Yes | No | Can’t tell |
| 1. Has the relationship between researcher and participants been adequately considered? | Yes | No | Can’t tell * |
| 1. Have ethical issues been taken into consideration? | Yes | No | Can’t tell |
| 1. Was the data analysis sufficiently rigorous? | Yes | No | Can’t tell |
| 1. Is there a clear statement of findings? | Yes | No | Can’t tell |
| 1. How valuable is the research? | Valuable | Not Valuable |  |

Result: INCLUDED *No mention of researcher’s own role, potential bias and influence.

**Modified CASP tool**

Reference: Manski-Nankervis J, Furler J, Blackberry I, Young D, O'Neal D, Patterson E: Roles and relationships between health professionals involved in insulin initiation for people with type 2 diabetes in the general practice setting: a qualitative study drawing on relational coordination theory. *BMC Family Practice* 2014, 15:1-10.

| 1. Was there a clear statement of the aims of the research? | Yes | No | Can’t tell |
| --- | --- | --- | --- |
| 1. Is a qualitative methodology appropriate? | Yes | No | Can’t tell |
| 1. Was there specific mention of advanced practice nursing (per the definition provided in our paper)? | Yes | No | Can’t tell |
| 1. Was the research design appropriate to address the aims of the research? | Yes | No | Can’t tell |
| 1. Was the recruitment strategy appropriate to the aims of the research? | Yes | No | Can’t tell |
| 1. Was the data collected in a way that addressed the research issue? | Yes | No | Can’t tell |
| 1. Has the relationship between researcher and participants been adequately considered? | Yes | No | Can’t tell * |
| 1. Have ethical issues been taken into consideration? | Yes | No | Can’t tell |
| 1. Was the data analysis sufficiently rigorous? | Yes | No | Can’t tell |
| 1. Is there a clear statement of findings? | Yes | No | Can’t tell |
| 1. How valuable is the research? | Valuable | Not Valuable |  |

Result: INCLUDED *No mention of researcher’s own role, potential bias and influence.

**Modified CASP tool**

Reference: McKenna L, Halcomb E, Lane R, Zwar N: An investigation of barriers and enablers to advanced nursing roles in Australian general practice. *Collegian (Royal College of Nursing, Australia)* 2015, 22:183-189.

| 1. Was there a clear statement of the aims of the research? | Yes | No | Can’t tell |
| --- | --- | --- | --- |
| 1. Is a qualitative methodology appropriate? | Yes | No | Can’t tell |
| 1. Was there specific mention of advanced practice nursing (per the definition provided in our paper)? | Yes | No | Can’t tell |
| 1. Was the research design appropriate to address the aims of the research? | Yes | No | Can’t tell |
| 1. Was the recruitment strategy appropriate to the aims of the research? | Yes | No | Can’t tell |
| 1. Was the data collected in a way that addressed the research issue? | Yes | No | Can’t tell |
| 1. Has the relationship between researcher and participants been adequately considered? | Yes | No | Can’t tell * |
| 1. Have ethical issues been taken into consideration? | Yes | No | Can’t tell |
| 1. Was the data analysis sufficiently rigorous? | Yes | No | Can’t tell |
| 1. Is there a clear statement of findings? | Yes | No | Can’t tell |
| 1. How valuable is the research? | Valuable | Not Valuable |  |

Result: INCLUDED *No mention of researcher’s own role, potential bias and influence.

**Modified CASP tool**

Reference: McKinlay E, Garrett S, McBain L, Dowell T, Collings S, Stanley J: **New Zealand general practice nurses' roles in mental health care**. *International Nursing Review* 2011, **58**(2):225-233.

| 1. Was there a clear statement of the aims of the research? | Yes | No | Can’t tell |
| --- | --- | --- | --- |
| 1. Is a qualitative methodology appropriate? | Yes | No | Can’t tell |
| 1. Was there specific mention of advanced practice nursing (per the definition provided in our paper)? | Yes | No | Can’t tell |
| 1. Was the research design appropriate to address the aims of the research? | Yes | No | Can’t tell |
| 1. Was the recruitment strategy appropriate to the aims of the research? | Yes | No | Can’t tell ^1^ |
| 1. Was the data collected in a way that addressed the research issue? | Yes | No | Can’t tell |
| 1. Has the relationship between researcher and participants been adequately considered? | Yes | No | Can’t tell ^2^ |
| 1. Have ethical issues been taken into consideration? | Yes | No | Can’t tell |
| 1. Was the data analysis sufficiently rigorous? | Yes | No | Can’t tell |
| 1. Is there a clear statement of findings? | Yes | No | Can’t tell |
| 1. How valuable is the research? | Valuable | Not Valuable |  |

Result: INCLUDED 1. No explanation of how participants were selected. 2. No mention of researcher’s own role, potential bias and influence.

**Modified CASP tool**

Reference: Mills J, Chamberlain-Salaun J, Christie L, Kingston M, Gorman E, Harvey C: Australian nurses in general practice, enabling the provision of cervical screening and well women's health care services: a qualitative study. *BMC Nursing* 2012, 11(1):23-30.

| 1. Was there a clear statement of the aims of the research? | Yes | No | Can’t tell |
| --- | --- | --- | --- |
| 1. Is a qualitative methodology appropriate? | Yes | No | Can’t tell |
| 1. Was there specific mention of advanced practice nursing (per the definition provided in our paper)? | Yes | No | Can’t tell |
| 1. Was the research design appropriate to address the aims of the research? | Yes | No | Can’t tell |
| 1. Was the recruitment strategy appropriate to the aims of the research? | Yes | No | Can’t tell |
| 1. Was the data collected in a way that addressed the research issue? | Yes | No | Can’t tell |
| 1. Has the relationship between researcher and participants been adequately considered? | Yes | No | Can’t tell |
| 1. Have ethical issues been taken into consideration? | Yes | No | Can’t tell |
| 1. Was the data analysis sufficiently rigorous? | Yes | No | Can’t tell |
| 1. Is there a clear statement of findings? | Yes | No | Can’t tell |
| 1. How valuable is the research? | Valuable | Not Valuable |  |

Result: INCLUDED

**Modified CASP tool**

Reference: Mitchell J, Brown J, Smith C: Interprofessional education: a nurse practitioner impacts family medicine residents' smoking cessation counselling experiences. *Journal of Interprofessional Care* 2009, 23(4):401-409.

| 1. Was there a clear statement of the aims of the research? | Yes | No | Can’t tell |
| --- | --- | --- | --- |
| 1. Is a qualitative methodology appropriate? | Yes | No | Can’t tell |
| 1. Was there specific mention of advanced practice nursing (per the definition provided in our paper)? | Yes | No | Can’t tell |
| 1. Was the research design appropriate to address the aims of the research? | Yes | No | Can’t tell |
| 1. Was the recruitment strategy appropriate to the aims of the research? | Yes | No | Can’t tell |
| 1. Was the data collected in a way that addressed the research issue? | Yes | No | Can’t tell |
| 1. Has the relationship between researcher and participants been adequately considered? | Yes | No | Can’t tell ^1^ |
| 1. Have ethical issues been taken into consideration? | Yes | No | Can’t tell ^2^ |
| 1. Was the data analysis sufficiently rigorous? | Yes | No | Can’t tell |
| 1. Is there a clear statement of findings? | Yes | No | Can’t tell |
| 1. How valuable is the research? | Valuable | Not Valuable |  |

Result: INCLUDED 1. No mention of researcher’s own role, potential bias and influence. 2. No specific mention of ethics approval.

**Modified CASP tool**

Reference: Oandasan I, Hammond M, Conn L, Callahan S, Gallinaro A, Moaveni A: **Family practice registered nurses: The time has come**. *Canadian Family Physician* 2010, **56**(10):e375-e382.

| 1. Was there a clear statement of the aims of the research? | Yes | No | Can’t tell |
| --- | --- | --- | --- |
| 1. Is a qualitative methodology appropriate? | Yes | No | Can’t tell |
| 1. Was there specific mention of advanced practice nursing (per the definition provided in our paper)? | Yes | No | Can’t tell |
| 1. Was the research design appropriate to address the aims of the research? | Yes | No | Can’t tell |
| 1. Was the recruitment strategy appropriate to the aims of the research? | Yes | No | Can’t tell |
| 1. Was the data collected in a way that addressed the research issue? | Yes | No | Can’t tell |
| 1. Has the relationship between researcher and participants been adequately considered? | Yes | No | Can’t tell * |
| 1. Have ethical issues been taken into consideration? | Yes | No | Can’t tell |
| 1. Was the data analysis sufficiently rigorous? | Yes | No | Can’t tell |
| 1. Is there a clear statement of findings? | Yes | No | Can’t tell |
| 1. How valuable is the research? | Valuable | Not Valuable |  |

Result: INCLUDED *No mention of researcher’s own role, potential bias and influence.

**Modified CASP tool**

Reference: Phillips C, Dwan K, Pearce C, Hall S, Porritt J, Yates R, Sibbald B: Time to talk, time to see: changing microeconomies of professional practice among nurses and doctors in Australian general practice. *Contemporary Nurse* 2007, 26(1):136-144.

| 1. Was there a clear statement of the aims of the research? | Yes | No | Can’t tell |
| --- | --- | --- | --- |
| 1. Is a qualitative methodology appropriate? | Yes | No | Can’t tell |
| 1. Was there specific mention of advanced practice nursing (per the definition provided in our paper)? | Yes | No | Can’t tell |
| 1. Was the research design appropriate to address the aims of the research? | Yes | No | Can’t tell |
| 1. Was the recruitment strategy appropriate to the aims of the research? | Yes | No | Can’t tell |
| 1. Was the data collected in a way that addressed the research issue? | Yes | No | Can’t tell |
| 1. Has the relationship between researcher and participants been adequately considered? | Yes | No | Can’t tell * |
| 1. Have ethical issues been taken into consideration? | Yes | No | Can’t tell |
| 1. Was the data analysis sufficiently rigorous? | Yes | No | Can’t tell |
| 1. Is there a clear statement of findings? | Yes | No | Can’t tell |
| 1. How valuable is the research? | Valuable | Not Valuable |  |

Result: INCLUDED *No mention of researcher’s own role, potential bias and influence.

**Modified CASP tool**

Reference: Price A, Williams A: **Primary care nurse practitioners and the interface with secondary care: a qualitative study of referral practice**. *Journal of Interprofessional Care* 2003, **17**(3):239-250.

| 1. Was there a clear statement of the aims of the research? | Yes | No | Can’t tell |
| --- | --- | --- | --- |
| 1. Is a qualitative methodology appropriate? | Yes | No | Can’t tell |
| 1. Was there specific mention of advanced practice nursing (per the definition provided in our paper)? | Yes | No | Can’t tell |
| 1. Was the research design appropriate to address the aims of the research? | Yes | No | Can’t tell |
| 1. Was the recruitment strategy appropriate to the aims of the research? | Yes | No | Can’t tell |
| 1. Was the data collected in a way that addressed the research issue? | Yes | No | Can’t tell |
| 1. Has the relationship between researcher and participants been adequately considered? | Yes | No | Can’t tell ^1^ |
| 1. Have ethical issues been taken into consideration? | Yes | No | Can’t tell ^2^ |
| 1. Was the data analysis sufficiently rigorous? | Yes | No | Can’t tell |
| 1. Is there a clear statement of findings? | Yes | No | Can’t tell |
| 1. How valuable is the research? | Valuable | Not Valuable |  |

Result: INCLUDED 1. No mention of researcher’s own role, potential bias and influence. 2. No specific mention of ethics approval.

**Modified CASP tool**

Reference: Speed S, Luker K: **Getting a visit: how district nurses and general practitioners 'organise' each other in primary care**. *Sociology of Health and Illness* 2006, **28**(7):883-902.

| 1. Was there a clear statement of the aims of the research? | Yes | No | Can’t tell |
| --- | --- | --- | --- |
| 1. Is a qualitative methodology appropriate? | Yes | No | Can’t tell |
| 1. Was there specific mention of advanced practice nursing (per the definition provided in our paper)? | Yes | No | Can’t tell |
| 1. Was the research design appropriate to address the aims of the research? | Yes | No | Can’t tell |
| 1. Was the recruitment strategy appropriate to the aims of the research? | Yes | No | Can’t tell * |
| 1. Was the data collected in a way that addressed the research issue? | Yes | No | Can’t tell |
| 1. Has the relationship between researcher and participants been adequately considered? | Yes | No | Can’t tell |
| 1. Have ethical issues been taken into consideration? | Yes | No | Can’t tell |
| 1. Was the data analysis sufficiently rigorous? | Yes | No | Can’t tell |
| 1. Is there a clear statement of findings? | Yes | No | Can’t tell |
| 1. How valuable is the research? | Valuable | Not Valuable |  |

Result: INCLUDED * No explanation of how participants were selected.

**Modified CASP tool**

Reference: Sunaert P, Willems S, Feyen L, Bastiaens H, De Maeseneer J, Jenkins L, Nobels F, Samyn E, Vandekerckhove M, Wens J *et al*: Engaging GPs in insulin therapy initiation: a qualitative study evaluating a support program in the Belgian context. *BMC Family Practice* 2014, 15:1-9.

| 1. Was there a clear statement of the aims of the research? | Yes | No | Can’t tell |
| --- | --- | --- | --- |
| 1. Is a qualitative methodology appropriate? | Yes | No | Can’t tell |
| 1. Was there specific mention of advanced practice nursing (per the definition provided in our paper)? | Yes | No | Can’t tell |
| 1. Was the research design appropriate to address the aims of the research? | Yes | No | Can’t tell |
| 1. Was the recruitment strategy appropriate to the aims of the research? | Yes | No | Can’t tell |
| 1. Was the data collected in a way that addressed the research issue? | Yes | No | Can’t tell |
| 1. Has the relationship between researcher and participants been adequately considered? | Yes | No | Can’t tell * |
| 1. Have ethical issues been taken into consideration? | Yes | No | Can’t tell |
| 1. Was the data analysis sufficiently rigorous? | Yes | No | Can’t tell |
| 1. Is there a clear statement of findings? | Yes | No | Can’t tell |
| 1. How valuable is the research? | Valuable | Not Valuable |  |

Result: INCLUDED * No mention of researcher’s own role, potential bias and influence.

**Modified CASP tool**

Reference: Walsh A, Moore A, Barber A, Opsteen J: **Educational role of nurse practitioners in a family practice centre: perspectives of learners and nurses**. *Canadian Family Physician* 2014, **60**(6):e316-e321.

| 1. Was there a clear statement of the aims of the research? | Yes | No | Can’t tell |
| --- | --- | --- | --- |
| 1. Is a qualitative methodology appropriate? | Yes | No | Can’t tell |
| 1. Was there specific mention of advanced practice nursing (per the definition provided in our paper)? | Yes | No | Can’t tell |
| 1. Was the research design appropriate to address the aims of the research? | Yes | No | Can’t tell |
| 1. Was the recruitment strategy appropriate to the aims of the research? | Yes | No | Can’t tell |
| 1. Was the data collected in a way that addressed the research issue? | Yes | No | Can’t tell |
| 1. Has the relationship between researcher and participants been adequately considered? | Yes | No | Can’t tell |
| 1. Have ethical issues been taken into consideration? | Yes | No | Can’t tell |
| 1. Was the data analysis sufficiently rigorous? | Yes | No | Can’t tell |
| 1. Is there a clear statement of findings? | Yes | No | Can’t tell |
| 1. How valuable is the research? | Valuable | Not Valuable |  |

Result: INCLUDED
