# Supplementary material for: A systematic review of experiences of advanced practice nursing in general practice
Source: BMC Nurs. 2017 Jan 18;16:6. doi: 10.1186/s12912-016-0198-7 (PMC5241982; doi:10.1186/s12912-016-0198-7)
Supplement: Additional file 4: — Critical appraisal of excluded studies. Description of data: A consolidated document detailing the results of critical appraisal for excluded studies. (DOCX 80 kb) [file 12912_2016_198_MOESM4_ESM.docx]

# Additional file 4: Critical appraisal of excluded studies

**Modified CASP tool**

Reference: Breen A, Carr E, Mann E, Crossen-White H: **Acute back pain management in primary care: a qualitative pilot study of the feasibility of a nurse-led service in general practice**. *Journal of Nursing Management* 2004, **12**(3):201-209.

| 1. Was there a clear statement of the aims of the research? | Yes | No | Can’t tell |
| --- | --- | --- | --- |
| 1. Is a qualitative methodology appropriate? | Yes | No | Can’t tell |
| 1. Was there specific mention of advanced practice nursing (per the definition provided in our paper)? | Yes | No | Can’t tell |
| 1. Was the research design appropriate to address the aims of the research? | Yes | No | Can’t tell |
| 1. Was the recruitment strategy appropriate to the aims of the research? | Yes | No | Can’t tell |
| 1. Was the data collected in a way that addressed the research issue? | Yes | No | Can’t tell |
| 1. Has the relationship between researcher and participants been adequately considered? | Yes | No | Can’t tell |
| 1. Have ethical issues been taken into consideration? | Yes | No | Can’t tell |
| 1. Was the data analysis sufficiently rigorous? | Yes | No | Can’t tell |
| 1. Is there a clear statement of findings? | Yes | No | Can’t tell |
| 1. How valuable is the research? | Valuable | Not Valuable |  |

Result: EXCLUDED

**Modified CASP tool**

Reference: Geense W, van de Glind I, Visscher T, van Achterberg T: **Barriers, facilitators and attitudes influencing health promotion activities in general practice: an explorative pilot study**. *BMC Family Practice* 2013, **14**:1-10.

| 1. Was there a clear statement of the aims of the research? | Yes | No | Can’t tell |
| --- | --- | --- | --- |
| 1. Is a qualitative methodology appropriate? | Yes | No | Can’t tell |
| 1. Was there specific mention of advanced practice nursing (per the definition provided in our paper)? | Yes | No | Can’t tell |
| 1. Was the research design appropriate to address the aims of the research? | Yes | No | Can’t tell |
| 1. Was the recruitment strategy appropriate to the aims of the research? | Yes | No | Can’t tell |
| 1. Was the data collected in a way that addressed the research issue? | Yes | No | Can’t tell |
| 1. Has the relationship between researcher and participants been adequately considered? | Yes | No | Can’t tell |
| 1. Have ethical issues been taken into consideration? | Yes | No | Can’t tell |
| 1. Was the data analysis sufficiently rigorous? | Yes | No | Can’t tell |
| 1. Is there a clear statement of findings? | Yes | No | Can’t tell |
| 1. How valuable is the research? | Valuable | Not Valuable |  |

Result: EXCLUDED

**Modified CASP tool**

Reference: Hamirudin A, Charlton K, Walton K, Bonney A, Potter J, Milosavljevic M, Hodgkins A, Albert G, Ghosh A, Dalley A: **Feasibility of implementing routine nutritional screening for older adults in Australian general practices: a mixed-methods study**. *BMC Family Practice* 2014, **15**:1-9.

| 1. Was there a clear statement of the aims of the research? | Yes | No | Can’t tell |
| --- | --- | --- | --- |
| 1. Is a qualitative methodology appropriate? | Yes | No | Can’t tell |
| 1. Was there specific mention of advanced practice nursing (per the definition provided in our paper)? | Yes | No | Can’t tell |
| 1. Was the research design appropriate to address the aims of the research? | Yes | No | Can’t tell |
| 1. Was the recruitment strategy appropriate to the aims of the research? | Yes | No | Can’t tell |
| 1. Was the data collected in a way that addressed the research issue? | Yes | No | Can’t tell |
| 1. Has the relationship between researcher and participants been adequately considered? | Yes | No | Can’t tell |
| 1. Have ethical issues been taken into consideration? | Yes | No | Can’t tell |
| 1. Was the data analysis sufficiently rigorous? | Yes | No | Can’t tell |
| 1. Is there a clear statement of findings? | Yes | No | Can’t tell |
| 1. How valuable is the research? | Valuable | Not Valuable |  |

Result: EXCLUDED

**Modified CASP tool**

Reference: Hannon K, Lester H, Campbell S: **Patients' views of pay for performance in primary care: a qualitative study**. *British Journal of General Practice* 2012, **62**(598):e322-e328.

| 1. Was there a clear statement of the aims of the research? | Yes | No | Can’t tell |
| --- | --- | --- | --- |
| 1. Is a qualitative methodology appropriate? | Yes | No | Can’t tell |
| 1. Was there specific mention of advanced practice nursing (per the definition provided in our paper)? | Yes | No | Can’t tell |
| 1. Was the research design appropriate to address the aims of the research? | Yes | No | Can’t tell |
| 1. Was the recruitment strategy appropriate to the aims of the research? | Yes | No | Can’t tell |
| 1. Was the data collected in a way that addressed the research issue? | Yes | No | Can’t tell |
| 1. Has the relationship between researcher and participants been adequately considered? | Yes | No | Can’t tell |
| 1. Have ethical issues been taken into consideration? | Yes | No | Can’t tell |
| 1. Was the data analysis sufficiently rigorous? | Yes | No | Can’t tell |
| 1. Is there a clear statement of findings? | Yes | No | Can’t tell |
| 1. How valuable is the research? | Valuable | Not Valuable |  |

Result: EXCLUDED

**Modified CASP tool**

Reference: Infante F, Proudfoot J, Powell Davies G, Bubner T, Holton C, Beilby J, Harris M: **How people with chronic illnesses view their care in general practice: a qualitative study**. *Medical Journal of Australia* 2004, **181**(2):70-73.

| 1. Was there a clear statement of the aims of the research? | Yes | No | Can’t tell |
| --- | --- | --- | --- |
| 1. Is a qualitative methodology appropriate? | Yes | No | Can’t tell |
| 1. Was there specific mention of advanced practice nursing (per the definition provided in our paper)? | Yes | No | Can’t tell |
| 1. Was the research design appropriate to address the aims of the research? | Yes | No | Can’t tell |
| 1. Was the recruitment strategy appropriate to the aims of the research? | Yes | No | Can’t tell |
| 1. Was the data collected in a way that addressed the research issue? | Yes | No | Can’t tell |
| 1. Has the relationship between researcher and participants been adequately considered? | Yes | No | Can’t tell |
| 1. Have ethical issues been taken into consideration? | Yes | No | Can’t tell |
| 1. Was the data analysis sufficiently rigorous? | Yes | No | Can’t tell |
| 1. Is there a clear statement of findings? | Yes | No | Can’t tell |
| 1. How valuable is the research? | Valuable | Not Valuable |  |

Result: EXCLUDED

**Modified CASP tool**

Reference: Litchfield I, Bentham L, Lilford R, Greenfield S: **Test result communication in primary care: clinical and office staff perspectives**. *Family Practice* 2014, **31**(5):592-597.

| 1. Was there a clear statement of the aims of the research? | Yes | No | Can’t tell |
| --- | --- | --- | --- |
| 1. Is a qualitative methodology appropriate? | Yes | No | Can’t tell |
| 1. Was there specific mention of advanced practice nursing (per the definition provided in our paper)? | Yes | No | Can’t tell |
| 1. Was the research design appropriate to address the aims of the research? | Yes | No | Can’t tell |
| 1. Was the recruitment strategy appropriate to the aims of the research? | Yes | No | Can’t tell |
| 1. Was the data collected in a way that addressed the research issue? | Yes | No | Can’t tell |
| 1. Has the relationship between researcher and participants been adequately considered? | Yes | No | Can’t tell |
| 1. Have ethical issues been taken into consideration? | Yes | No | Can’t tell |
| 1. Was the data analysis sufficiently rigorous? | Yes | No | Can’t tell |
| 1. Is there a clear statement of findings? | Yes | No | Can’t tell |
| 1. How valuable is the research? | Valuable | Not Valuable |  |

Result: EXCLUDED

**Modified CASP tool**

Reference: Lorch R, Hocking J, Guy R, Vaisey A, Wood A, Donovan B, Fairley C, Gunn J, Kaldor J, Temple-Smith M: **Do Australian general practitioners believe practice nurses can take a role in chlamydia testing? A qualitative study of attitudes and opinions**. *BMC Infectious Diseases* 2015, **15**:1-8.

| 1. Was there a clear statement of the aims of the research? | Yes | No | Can’t tell |
| --- | --- | --- | --- |
| 1. Is a qualitative methodology appropriate? | Yes | No | Can’t tell |
| 1. Was there specific mention of advanced practice nursing (per the definition provided in our paper)? | Yes | No | Can’t tell |
| 1. Was the research design appropriate to address the aims of the research? | Yes | No | Can’t tell |
| 1. Was the recruitment strategy appropriate to the aims of the research? | Yes | No | Can’t tell |
| 1. Was the data collected in a way that addressed the research issue? | Yes | No | Can’t tell |
| 1. Has the relationship between researcher and participants been adequately considered? | Yes | No | Can’t tell |
| 1. Have ethical issues been taken into consideration? | Yes | No | Can’t tell |
| 1. Was the data analysis sufficiently rigorous? | Yes | No | Can’t tell |
| 1. Is there a clear statement of findings? | Yes | No | Can’t tell |
| 1. How valuable is the research? | Valuable | Not Valuable |  |

Result: EXCLUDED

**Modified CASP tool**

Reference: McLaren S, Woods L, Boudioni M, Lemma F, Rees S, Broadbent J: **Developing the general practice manager role: managers' experiences of engagement in continuing professional development**. *Quality in Primary Care* 2007, **15**(2):85-91.

| 1. Was there a clear statement of the aims of the research? | Yes | No | Can’t tell |
| --- | --- | --- | --- |
| 1. Is a qualitative methodology appropriate? | Yes | No | Can’t tell |
| 1. Was there specific mention of advanced practice nursing (per the definition provided in our paper)? | Yes | No | Can’t tell |
| 1. Was the research design appropriate to address the aims of the research? | Yes | No | Can’t tell |
| 1. Was the recruitment strategy appropriate to the aims of the research? | Yes | No | Can’t tell |
| 1. Was the data collected in a way that addressed the research issue? | Yes | No | Can’t tell |
| 1. Has the relationship between researcher and participants been adequately considered? | Yes | No | Can’t tell |
| 1. Have ethical issues been taken into consideration? | Yes | No | Can’t tell |
| 1. Was the data analysis sufficiently rigorous? | Yes | No | Can’t tell |
| 1. Is there a clear statement of findings? | Yes | No | Can’t tell |
| 1. How valuable is the research? | Valuable | Not Valuable |  |

Result: EXCLUDED

**Modified CASP tool**

Reference: Merrick E, Fry M, Duffield C, Stasa H: **Trust and decision-making: How nurses in Australian general practice negotiate role limitations**. *Collegian (Royal College of Nursing, Australia)* 2015, **22**(2):225-232.

| 1. Was there a clear statement of the aims of the research? | Yes | No | Can’t tell |
| --- | --- | --- | --- |
| 1. Is a qualitative methodology appropriate? | Yes | No | Can’t tell |
| 1. Was there specific mention of advanced practice nursing (per the definition provided in our paper)? | Yes | No | Can’t tell |
| 1. Was the research design appropriate to address the aims of the research? | Yes | No | Can’t tell |
| 1. Was the recruitment strategy appropriate to the aims of the research? | Yes | No | Can’t tell |
| 1. Was the data collected in a way that addressed the research issue? | Yes | No | Can’t tell |
| 1. Has the relationship between researcher and participants been adequately considered? | Yes | No | Can’t tell |
| 1. Have ethical issues been taken into consideration? | Yes | No | Can’t tell |
| 1. Was the data analysis sufficiently rigorous? | Yes | No | Can’t tell |
| 1. Is there a clear statement of findings? | Yes | No | Can’t tell |
| 1. How valuable is the research? | Valuable | Not Valuable |  |

Result: EXCLUDED

**Modified CASP tool**

Reference: Moffat M, Cleland J, van der Molen T, Price D: **Poor communication may impair optimal asthma care: a qualitative study**. *Family Practice* 2007, **24**(1):65-70.

| 1. Was there a clear statement of the aims of the research? | Yes | No | Can’t tell |
| --- | --- | --- | --- |
| 1. Is a qualitative methodology appropriate? | Yes | No | Can’t tell |
| 1. Was there specific mention of advanced practice nursing (per the definition provided in our paper)? | Yes | No | Can’t tell |
| 1. Was the research design appropriate to address the aims of the research? | Yes | No | Can’t tell |
| 1. Was the recruitment strategy appropriate to the aims of the research? | Yes | No | Can’t tell |
| 1. Was the data collected in a way that addressed the research issue? | Yes | No | Can’t tell |
| 1. Has the relationship between researcher and participants been adequately considered? | Yes | No | Can’t tell |
| 1. Have ethical issues been taken into consideration? | Yes | No | Can’t tell |
| 1. Was the data analysis sufficiently rigorous? | Yes | No | Can’t tell |
| 1. Is there a clear statement of findings? | Yes | No | Can’t tell |
| 1. How valuable is the research? | Valuable | Not Valuable |  |

Result: EXCLUDED

**Modified CASP tool**

Reference: Noordman J, Koopmans B, Korevaar J, van der Weijden T, van Dulmen S: **Exploring lifestyle counselling in routine primary care consultations: the professionals' role**. *Family Practice* 2013, **30**(3):332-340.

| 1. Was there a clear statement of the aims of the research? | Yes | No | Can’t tell |
| --- | --- | --- | --- |
| 1. Is a qualitative methodology appropriate? | Yes | No | Can’t tell |
| 1. Was there specific mention of advanced practice nursing (per the definition provided in our paper)? | Yes | No | Can’t tell |
| 1. Was the research design appropriate to address the aims of the research? | Yes | No | Can’t tell |
| 1. Was the recruitment strategy appropriate to the aims of the research? | Yes | No | Can’t tell |
| 1. Was the data collected in a way that addressed the research issue? | Yes | No | Can’t tell |
| 1. Has the relationship between researcher and participants been adequately considered? | Yes | No | Can’t tell |
| 1. Have ethical issues been taken into consideration? | Yes | No | Can’t tell |
| 1. Was the data analysis sufficiently rigorous? | Yes | No | Can’t tell |
| 1. Is there a clear statement of findings? | Yes | No | Can’t tell |
| 1. How valuable is the research? | Valuable | Not Valuable |  |

Result: EXCLUDED

**Modified CASP tool**

Reference: Ocek Z, Ciceklioglu M, Yucel U, Ozdemir R: **Family medicine model in Turkey: a qualitative assessment from the perspectives of primary care workers**. *BMC Family Practice* 2014, **15**:1-15.

| 1. Was there a clear statement of the aims of the research? | Yes | No | Can’t tell |
| --- | --- | --- | --- |
| 1. Is a qualitative methodology appropriate? | Yes | No | Can’t tell |
| 1. Was there specific mention of advanced practice nursing (per the definition provided in our paper)? | Yes | No | Can’t tell |
| 1. Was the research design appropriate to address the aims of the research? | Yes | No | Can’t tell |
| 1. Was the recruitment strategy appropriate to the aims of the research? | Yes | No | Can’t tell |
| 1. Was the data collected in a way that addressed the research issue? | Yes | No | Can’t tell |
| 1. Has the relationship between researcher and participants been adequately considered? | Yes | No | Can’t tell |
| 1. Have ethical issues been taken into consideration? | Yes | No | Can’t tell |
| 1. Was the data analysis sufficiently rigorous? | Yes | No | Can’t tell |
| 1. Is there a clear statement of findings? | Yes | No | Can’t tell |
| 1. How valuable is the research? | Valuable | Not Valuable |  |

Result: EXCLUDED

**Modified CASP tool**

Reference: Pearce C, Hall S, Phillips C, Dwan K, Yates R, Sibbald B: **A spatial analysis of the expanding roles of nurses in general practice**. *BMC Nursing* 2012, **11**(1):13-20.

| 1. Was there a clear statement of the aims of the research? | Yes | No | Can’t tell |
| --- | --- | --- | --- |
| 1. Is a qualitative methodology appropriate? | Yes | No | Can’t tell |
| 1. Was there specific mention of advanced practice nursing (per the definition provided in our paper)? | Yes | No | Can’t tell |
| 1. Was the research design appropriate to address the aims of the research? | Yes | No | Can’t tell |
| 1. Was the recruitment strategy appropriate to the aims of the research? | Yes | No | Can’t tell |
| 1. Was the data collected in a way that addressed the research issue? | Yes | No | Can’t tell |
| 1. Has the relationship between researcher and participants been adequately considered? | Yes | No | Can’t tell |
| 1. Have ethical issues been taken into consideration? | Yes | No | Can’t tell |
| 1. Was the data analysis sufficiently rigorous? | Yes | No | Can’t tell |
| 1. Is there a clear statement of findings? | Yes | No | Can’t tell |
| 1. How valuable is the research? | Valuable | Not Valuable |  |

Result: EXCLUDED

**Modified CASP tool**

Reference: Reay T, Goodrick E, Casebeer A, Hinings C: **Legitimizing new practices in primary health care**. *Health Care Management Review* 2013, **38**(1):9-19.

| 1. Was there a clear statement of the aims of the research? | Yes | No | Can’t tell |
| --- | --- | --- | --- |
| 1. Is a qualitative methodology appropriate? | Yes | No | Can’t tell |
| 1. Was there specific mention of advanced practice nursing (per the definition provided in our paper)? | Yes | No | Can’t tell |
| 1. Was the research design appropriate to address the aims of the research? | Yes | No | Can’t tell |
| 1. Was the recruitment strategy appropriate to the aims of the research? | Yes | No | Can’t tell |
| 1. Was the data collected in a way that addressed the research issue? | Yes | No | Can’t tell |
| 1. Has the relationship between researcher and participants been adequately considered? | Yes | No | Can’t tell |
| 1. Have ethical issues been taken into consideration? | Yes | No | Can’t tell |
| 1. Was the data analysis sufficiently rigorous? | Yes | No | Can’t tell |
| 1. Is there a clear statement of findings? | Yes | No | Can’t tell |
| 1. How valuable is the research? | Valuable | Not Valuable |  |

Result: EXCLUDED

**Modified CASP tool**

Reference: Redsell S, Jackson C, Stokes T, Hastings A, Baker R: **Patient expectations of 'first-contact care' consultations with nurse and general practitioners in primary care**. *Quality in Primary Care* 2007, **15**(1):5-10.

| 1. Was there a clear statement of the aims of the research? | Yes | No | Can’t tell |
| --- | --- | --- | --- |
| 1. Is a qualitative methodology appropriate? | Yes | No | Can’t tell |
| 1. Was there specific mention of advanced practice nursing (per the definition provided in our paper)? | Yes | No | Can’t tell |
| 1. Was the research design appropriate to address the aims of the research? | Yes | No | Can’t tell |
| 1. Was the recruitment strategy appropriate to the aims of the research? | Yes | No | Can’t tell |
| 1. Was the data collected in a way that addressed the research issue? | Yes | No | Can’t tell |
| 1. Has the relationship between researcher and participants been adequately considered? | Yes | No | Can’t tell |
| 1. Have ethical issues been taken into consideration? | Yes | No | Can’t tell |
| 1. Was the data analysis sufficiently rigorous? | Yes | No | Can’t tell |
| 1. Is there a clear statement of findings? | Yes | No | Can’t tell |
| 1. How valuable is the research? | Valuable | Not Valuable |  |

Result: EXCLUDED

**Modified CASP tool**

Reference: Reinders M, Blankenstein A, van Marwijk H, Schleypen H, Schoonheim P, Stalman W: **Development and feasibility of a patient feedback programme to improve consultation skills in general practice training**. *Patient Education and Counselling* 2008, **72**(1):12-19.

| 1. Was there a clear statement of the aims of the research? | Yes | No | Can’t tell |
| --- | --- | --- | --- |
| 1. Is a qualitative methodology appropriate? | Yes | No | Can’t tell |
| 1. Was there specific mention of advanced practice nursing (per the definition provided in our paper)? | Yes | No | Can’t tell |
| 1. Was the research design appropriate to address the aims of the research? | Yes | No | Can’t tell |
| 1. Was the recruitment strategy appropriate to the aims of the research? | Yes | No | Can’t tell |
| 1. Was the data collected in a way that addressed the research issue? | Yes | No | Can’t tell |
| 1. Has the relationship between researcher and participants been adequately considered? | Yes | No | Can’t tell |
| 1. Have ethical issues been taken into consideration? | Yes | No | Can’t tell |
| 1. Was the data analysis sufficiently rigorous? | Yes | No | Can’t tell |
| 1. Is there a clear statement of findings? | Yes | No | Can’t tell |
| 1. How valuable is the research? | Valuable | Not Valuable |  |

Result: EXCLUDED

**Modified CASP tool**

Reference: Rodriguez C, Pozzebon M: **The implementation evaluation of primary care groups of practice: a focus on organizational identity**. *BMC Family Practice* 2010, **11**:1-10.

| 1. Was there a clear statement of the aims of the research? | Yes | No | Can’t tell |
| --- | --- | --- | --- |
| 1. Is a qualitative methodology appropriate? | Yes | No | Can’t tell |
| 1. Was there specific mention of advanced practice nursing (per the definition provided in our paper)? | Yes | No | Can’t tell |
| 1. Was the research design appropriate to address the aims of the research? | Yes | No | Can’t tell |
| 1. Was the recruitment strategy appropriate to the aims of the research? | Yes | No | Can’t tell |
| 1. Was the data collected in a way that addressed the research issue? | Yes | No | Can’t tell |
| 1. Has the relationship between researcher and participants been adequately considered? | Yes | No | Can’t tell |
| 1. Have ethical issues been taken into consideration? | Yes | No | Can’t tell |
| 1. Was the data analysis sufficiently rigorous? | Yes | No | Can’t tell |
| 1. Is there a clear statement of findings? | Yes | No | Can’t tell |
| 1. How valuable is the research? | Valuable | Not Valuable |  |

Result: EXCLUDED

**Modified CASP tool**

Reference: Rosemann T, Joest K, Korner T, Schaefert R, Heiderhoff M, Szecsenyi J: **How can the practice nurse be more involved in the care of the chronically ill? The perspectives of GPs, patients and practice nurses**. *BMC Family Practice* 2006, **7**:1-7.

| 1. Was there a clear statement of the aims of the research? | Yes | No | Can’t tell |
| --- | --- | --- | --- |
| 1. Is a qualitative methodology appropriate? | Yes | No | Can’t tell |
| 1. Was there specific mention of advanced practice nursing (per the definition provided in our paper)? | Yes | No | Can’t tell |
| 1. Was the research design appropriate to address the aims of the research? | Yes | No | Can’t tell |
| 1. Was the recruitment strategy appropriate to the aims of the research? | Yes | No | Can’t tell |
| 1. Was the data collected in a way that addressed the research issue? | Yes | No | Can’t tell |
| 1. Has the relationship between researcher and participants been adequately considered? | Yes | No | Can’t tell |
| 1. Have ethical issues been taken into consideration? | Yes | No | Can’t tell |
| 1. Was the data analysis sufficiently rigorous? | Yes | No | Can’t tell |
| 1. Is there a clear statement of findings? | Yes | No | Can’t tell |
| 1. How valuable is the research? | Valuable | Not Valuable |  |

Result: EXCLUDED

**Modified CASP tool**

Reference: Rosenberg E, Richard C, Lussier M, Abdool S: **Intercultural communication competence in family medicine: lessons from the field**. *Patient Education and Counselling* 2006, **61**(2):236-245.

| 1. Was there a clear statement of the aims of the research? | Yes | No | Can’t tell |
| --- | --- | --- | --- |
| 1. Is a qualitative methodology appropriate? | Yes | No | Can’t tell |
| 1. Was there specific mention of advanced practice nursing (per the definition provided in our paper)? | Yes | No | Can’t tell |
| 1. Was the research design appropriate to address the aims of the research? | Yes | No | Can’t tell |
| 1. Was the recruitment strategy appropriate to the aims of the research? | Yes | No | Can’t tell |
| 1. Was the data collected in a way that addressed the research issue? | Yes | No | Can’t tell |
| 1. Has the relationship between researcher and participants been adequately considered? | Yes | No | Can’t tell |
| 1. Have ethical issues been taken into consideration? | Yes | No | Can’t tell |
| 1. Was the data analysis sufficiently rigorous? | Yes | No | Can’t tell |
| 1. Is there a clear statement of findings? | Yes | No | Can’t tell |
| 1. How valuable is the research? | Valuable | Not Valuable |  |

Result: EXCLUDED

**Modified CASP tool**

Reference: Rousseau N, McColl E, Newton J, Grimshaw J, Eccles M: **Practice based, longitudinal, qualitative interview study of computerised evidence based guidelines in primary care**. *British Medical Journal* 2003, **326**(7384):1-8.

| 1. Was there a clear statement of the aims of the research? | Yes | No | Can’t tell |
| --- | --- | --- | --- |
| 1. Is a qualitative methodology appropriate? | Yes | No | Can’t tell |
| 1. Was there specific mention of advanced practice nursing (per the definition provided in our paper)? | Yes | No | Can’t tell |
| 1. Was the research design appropriate to address the aims of the research? | Yes | No | Can’t tell |
| 1. Was the recruitment strategy appropriate to the aims of the research? | Yes | No | Can’t tell |
| 1. Was the data collected in a way that addressed the research issue? | Yes | No | Can’t tell |
| 1. Has the relationship between researcher and participants been adequately considered? | Yes | No | Can’t tell |
| 1. Have ethical issues been taken into consideration? | Yes | No | Can’t tell |
| 1. Was the data analysis sufficiently rigorous? | Yes | No | Can’t tell |
| 1. Is there a clear statement of findings? | Yes | No | Can’t tell |
| 1. How valuable is the research? | Valuable | Not Valuable |  |

Result: EXCLUDED

**Modified CASP tool**

Reference: Slight S, Howard R, Ghaleb M, Barber N, Franklin B, Avery A: **The causes of prescribing errors in English general practices: a qualitative study**. *British Journal of General Practice* 2013, **63**(615):e713-e720.

| 1. Was there a clear statement of the aims of the research? | Yes | No | Can’t tell |
| --- | --- | --- | --- |
| 1. Is a qualitative methodology appropriate? | Yes | No | Can’t tell |
| 1. Was there specific mention of advanced practice nursing (per the definition provided in our paper)? | Yes | No | Can’t tell |
| 1. Was the research design appropriate to address the aims of the research? | Yes | No | Can’t tell |
| 1. Was the recruitment strategy appropriate to the aims of the research? | Yes | No | Can’t tell |
| 1. Was the data collected in a way that addressed the research issue? | Yes | No | Can’t tell |
| 1. Has the relationship between researcher and participants been adequately considered? | Yes | No | Can’t tell |
| 1. Have ethical issues been taken into consideration? | Yes | No | Can’t tell |
| 1. Was the data analysis sufficiently rigorous? | Yes | No | Can’t tell |
| 1. Is there a clear statement of findings? | Yes | No | Can’t tell |
| 1. How valuable is the research? | Valuable | Not Valuable |  |

Result: EXCLUDED

**Modified CASP tool**

Reference: van Rossem C, Spigt M, Kleijsen J, Hendricx M, van Schayck C, Kotz D: **Smoking cessation in primary care: Exploration of barriers and solutions in current daily practice from the perspective of smokers and healthcare professionals**. *European Journal of General Practice* 2015, **21**(2):111-117.

| 1. Was there a clear statement of the aims of the research? | Yes | No | Can’t tell |
| --- | --- | --- | --- |
| 1. Is a qualitative methodology appropriate? | Yes | No | Can’t tell |
| 1. Was there specific mention of advanced practice nursing (per the definition provided in our paper)? | Yes | No | Can’t tell |
| 1. Was the research design appropriate to address the aims of the research? | Yes | No | Can’t tell |
| 1. Was the recruitment strategy appropriate to the aims of the research? | Yes | No | Can’t tell |
| 1. Was the data collected in a way that addressed the research issue? | Yes | No | Can’t tell |
| 1. Has the relationship between researcher and participants been adequately considered? | Yes | No | Can’t tell |
| 1. Have ethical issues been taken into consideration? | Yes | No | Can’t tell |
| 1. Was the data analysis sufficiently rigorous? | Yes | No | Can’t tell |
| 1. Is there a clear statement of findings? | Yes | No | Can’t tell |
| 1. How valuable is the research? | Valuable | Not Valuable |  |

Result: EXCLUDED

**Modified CASP tool**

Reference: Wiles R: **Empowering practice nurses in the follow-up of patients with established heart disease: lessons from patients' experiences**. *Journal of Advanced Nursing* 1997, **26**(4):729-735.

| 1. Was there a clear statement of the aims of the research? | Yes | No | Can’t tell |
| --- | --- | --- | --- |
| 1. Is a qualitative methodology appropriate? | Yes | No | Can’t tell |
| 1. Was there specific mention of advanced practice nursing (per the definition provided in our paper)? | Yes | No | Can’t tell |
| 1. Was the research design appropriate to address the aims of the research? | Yes | No | Can’t tell |
| 1. Was the recruitment strategy appropriate to the aims of the research? | Yes | No | Can’t tell |
| 1. Was the data collected in a way that addressed the research issue? | Yes | No | Can’t tell |
| 1. Has the relationship between researcher and participants been adequately considered? | Yes | No | Can’t tell |
| 1. Have ethical issues been taken into consideration? | Yes | No | Can’t tell |
| 1. Was the data analysis sufficiently rigorous? | Yes | No | Can’t tell |
| 1. Is there a clear statement of findings? | Yes | No | Can’t tell |
| 1. How valuable is the research? | Valuable | Not Valuable |  |

Result: EXCLUDED

**Modified CASP tool**

Reference: Woodcock H, Gillam S: **A one-to-one thing is better than a thousand books': views and understanding of older people with diabetes**. *Quality in Primary Care* 2013, **21**(3):157-163.

| 1. Was there a clear statement of the aims of the research? | Yes | No | Can’t tell |
| --- | --- | --- | --- |
| 1. Is a qualitative methodology appropriate? | Yes | No | Can’t tell |
| 1. Was there specific mention of advanced practice nursing (per the definition provided in our paper)? | Yes | No | Can’t tell |
| 1. Was the research design appropriate to address the aims of the research? | Yes | No | Can’t tell |
| 1. Was the recruitment strategy appropriate to the aims of the research? | Yes | No | Can’t tell |
| 1. Was the data collected in a way that addressed the research issue? | Yes | No | Can’t tell |
| 1. Has the relationship between researcher and participants been adequately considered? | Yes | No | Can’t tell |
| 1. Have ethical issues been taken into consideration? | Yes | No | Can’t tell |
| 1. Was the data analysis sufficiently rigorous? | Yes | No | Can’t tell |
| 1. Is there a clear statement of findings? | Yes | No | Can’t tell |
| 1. How valuable is the research? | Valuable | Not Valuable |  |

Result: EXCLUDED

**Modified CASP tool**

Reference: Wright F, Wiles R, Moher M: **Patients' and practice nurses' perceptions of secondary preventive care for established ischaemic heart disease: a qualitative study**. *Journal of Clinical Nursing* 2001, **10**(2):180-188.

| 1. Was there a clear statement of the aims of the research? | Yes | No | Can’t tell |
| --- | --- | --- | --- |
| 1. Is a qualitative methodology appropriate? | Yes | No | Can’t tell |
| 1. Was there specific mention of advanced practice nursing (per the definition provided in our paper)? | Yes | No | Can’t tell |
| 1. Was the research design appropriate to address the aims of the research? | Yes | No | Can’t tell |
| 1. Was the recruitment strategy appropriate to the aims of the research? | Yes | No | Can’t tell |
| 1. Was the data collected in a way that addressed the research issue? | Yes | No | Can’t tell |
| 1. Has the relationship between researcher and participants been adequately considered? | Yes | No | Can’t tell |
| 1. Have ethical issues been taken into consideration? | Yes | No | Can’t tell |
| 1. Was the data analysis sufficiently rigorous? | Yes | No | Can’t tell |
| 1. Is there a clear statement of findings? | Yes | No | Can’t tell |
| 1. How valuable is the research? | Valuable | Not Valuable |  |

Result: EXCLUDED
