# Supplementary material for: A systematic review of experiences of advanced practice nursing in general practice
Source: BMC Nurs. 2017 Jan 18;16:6. doi: 10.1186/s12912-016-0198-7 (PMC5241982; doi:10.1186/s12912-016-0198-7)
Supplement: Additional file 5 — List of studies contributing to each code and sub-theme. Description of data: A table listing the studies that contributed to each code and sub-theme. (DOCX 20 kb) [file 12912_2016_198_MOESM5_ESM.docx]

# Additional file 5: List of studies contributing to each code and sub-theme

| Major Theme | Sub-themes | Codes | Studies containing each code |
| --- | --- | --- | --- |
|  | Establishing (E) and Maintaining (M) confidence in the advanced practice nurse | Accountability (M)  Developing (E)  Feedback (M)  Referring (M)  Relinquishing (E)  Respecting (M)  Responsibility (E, M)  Trusting (E, M) | [8-10, 27, 28, 30, 33, 36]  [8-10, 22-30, 34, 35]  [8, 15, 33, 35, 36]  [27-30, 36]  [8-10, 15, 24, 25, 28, 30, 31, 36]  [8, 15, 25, 27, 29-32, 34-36]  [8, 10, 15, 23, 28, 30, 32, 33]  [8-10, 15, 23, 25, 28, 30-32, 34-36] |
| Legitimacy | Strengthening (S) and Weakening (W) boundaries between general practitioners and advanced practice nurses | Advocating (W)  Ambiguity (S)  Clarifying (W)  Collaborating (W)  Expanding (W)  Flexibility (W)  Negotiating (W)  Protocolling (S)  Reforming (W)  Sharing (W)  Spanning (W)  Traditionalising (S) | [31]  [15, 27, 30, 31]  [10, 15, 28, 30, 31, 34, 36]  [10, 25-28, 30-32, 34, 35]  [8, 9, 23, 25, 26, 28, 30, 31, 34, 35]  [15, 23, 27, 28, 37]  [8, 9, 10, 15, 23, 28, 30, 32, 34, 35, 36]  [9, 10, 15, 25, 27, 28, 31-36]  [8, 9, 25, 26, 28, 31, 32, 35]  [8, 25, 26, 32-34]  [10, 27, 32, 34]  [10, 15, 25, 27, 28, 30-32, 34, 35, 37] |
|  | Establishing (E) and Maintaining (M) the value of advanced practice nursing | Costing (E)  Funding (M)  Timing (M)  Resources (E, M)  Continuity (M)  Availability (M)  Targeting (E) | [9, 10, 24, 25, 30, 31, 33, 34, 37]  [9, 24, 31, 32, 34]  [8-10, 22, 23, 25-27, 30-37]  [15, 24-28, 30, 32, 34, 35, 37]  [8, 9, 23-25, 28, 30-32, 34]  [15, 22, 27, 28, 31, 32, 34, 37]  [10, 15, 25-28, 32, 34, 36] |
